# Supplementary material for: Structure of PDE3A–SLFN12 complex and structure-based design for a potent apoptosis inducer of tumor cells
Source: Nat Commun. 2021 Oct 27;12:6204. doi: 10.1038/s41467-021-26546-8 (PMC8551160; doi:10.1038/s41467-021-26546-8)
Supplement: Supplementary file 1 — Supplementary Information [file 41467_2021_26546_MOESM1_ESM.pdf]

Supplementary Information

Structure of PDE3A–SLFN12 Complex and Structure-based Design for A potent Apoptosis inducer of Tumor Cells

Chen *et al*.

Supplementary Figure 1-9

Supplementary Table1-4

Supplementary Note 1

Supplementary Methods

Supplementary Figures

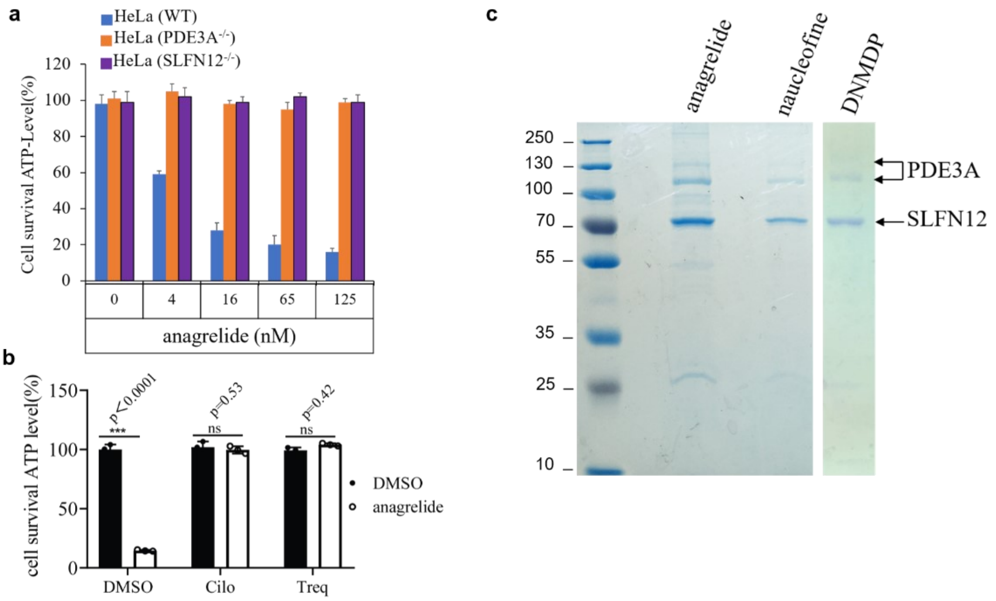

Supplementary Figure 1. The structurally diverse chemicals induce apoptosis by forming a complex with PDE3A and SLFN12. (a) HeLa cells were treated with the indicated stimuli for 36 hours. Cell viability was determined by measuring ATP levels (n=3, examined in two independent experiments). The data are represented as the mean ± SD of triplicate wells. Identical concentrations of anagrelide (100 nM), cilostazol (1 μM) and trequinsin (25 nM) were used in subsequent experiments unless otherwise stated. Ana, anagrelide; Cilo, cilostazol; Treq, trequinsin. (b) HeLa (WT), HeLa (PDE3A$^{-/-}$) and HeLa (SLFN12$^{-/-}$) cells were treated with anagrelide with the indicated concentration for 36 hours. Cell viability was determined by measuring ATP levels (n=3, examined in two independent experiments). The data are represented as the mean ± SD of triplicate wells. Student's t test (two-tailed, unpaired) was performed, ns, not significant, ***p<0.001. (c) HeLa-SLFN12 (K213R) -HA-3×Flag cells were treated with the indicated stimuli for 12 hr. The cell lysates were used for anti-Flag and anti-HA tandem pull-down. Polyacrylamide gels were stained with Coomassie brilliant blue according to the standard staining protocols. This is a representative result from two independent experiments.

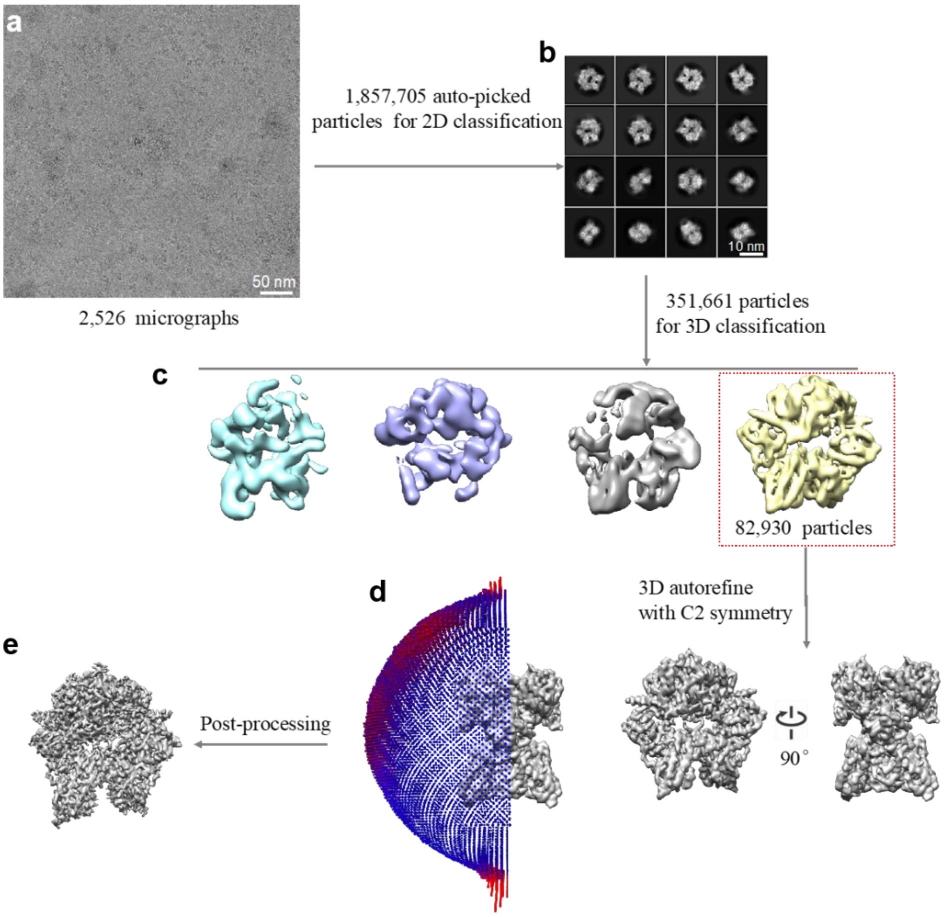

Supplementary Figure 2. Flowchart for the cryo-EM data processing of anagrelide-induced PDE3A-SLFN12 complex. Structures of DNMDP- and nauclefine-induced PDE3A-SLFN12 complexes were determined by the same flowchart (Details in Method section). (a) A representative cryo-EM micrograph of the PDE3A-SLFN12 complex. (b) The 2D classification results. (c) The 3D classification results. (d) 3D auto-refinement with C2 symmetry. The angular distribution was displayed. (e) The sharpened density of the PDE3A-SLFN12 complex.

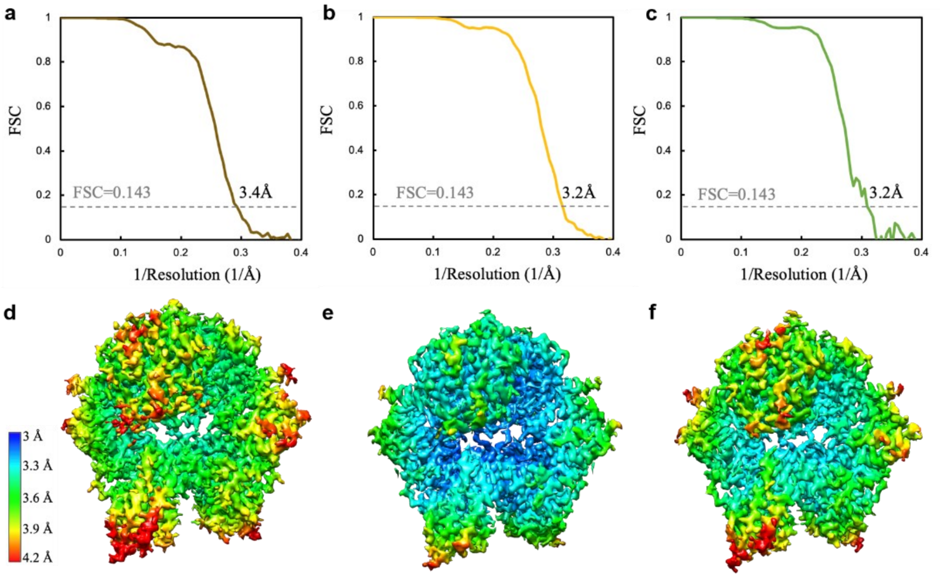

Supplementary Figure 3. Cryo-EM reconstruction results of anagrelide-, DNMDP and nauclefine-induced PDE3A-SLFN12 complexes. (a-c) The FSC curves of the reconstructions of anagrelide- (a), DNMDP- (b) and nauclefine- (c) induced PDE3A-SLFN12 complexes. FSC=0.143 was indicated by dotted lines. (d-f) Local resolution distribution of the cryo-EM densities of anagrelide- (d), DNMDP- (e) and nauclefine- (f) induced PDE3A-SLFN12 complexes.

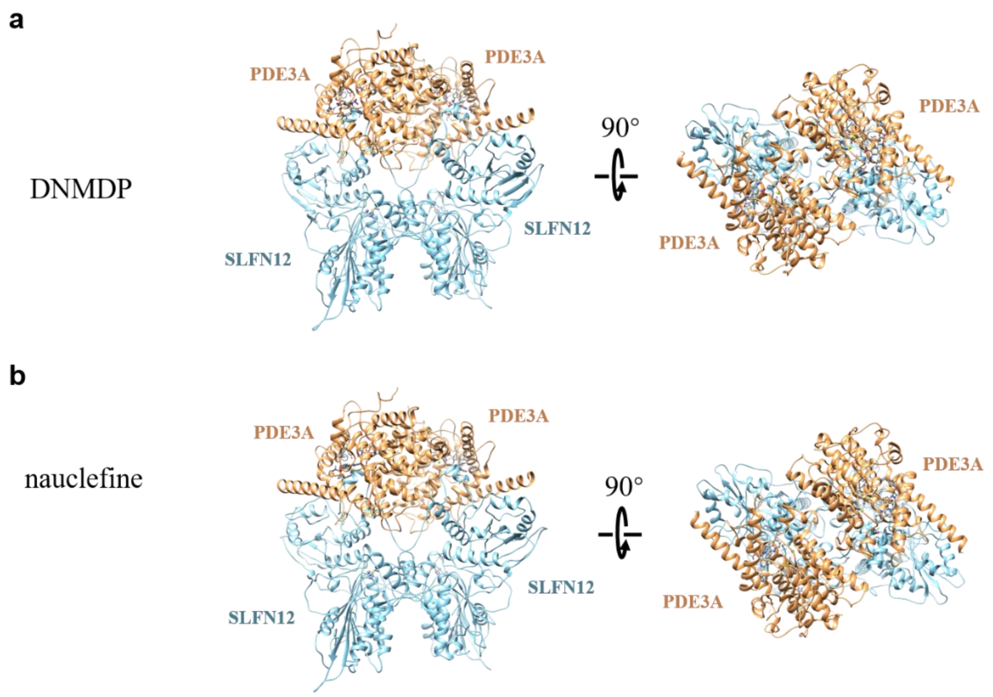

Supplementary Figure 4. Structures of DNMDP- (a) and nauclefine- (b) induced PDE3A-SLFN12 complex, with PDE3A colored in sandy brown and SLFN12 in light blue.

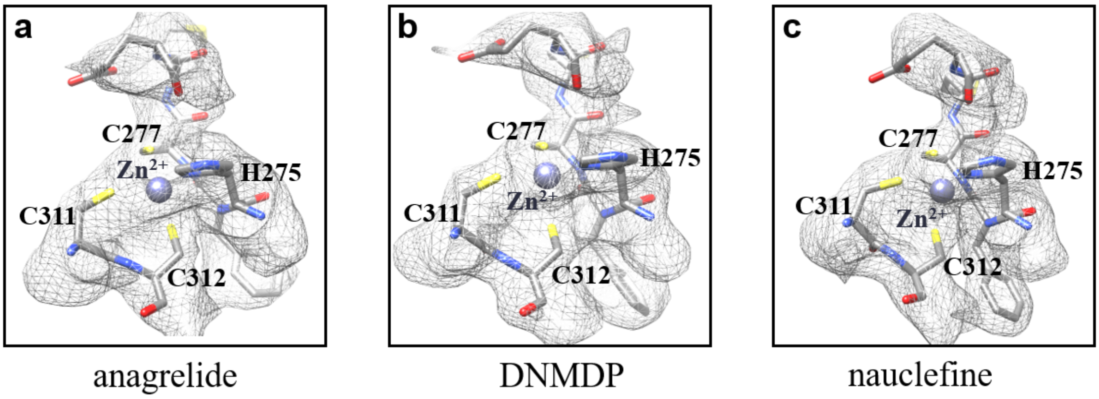

Supplementary Figure 5. Cryo-EM Densities of the zinc finger. Cryo-EM Densities of zinc fingers of anagrelide- (a), DNMDP- (b) and nauclefine- (c) induced PDE3A-SLFN12. All these densities were shown with a contour level of mean+60σ.

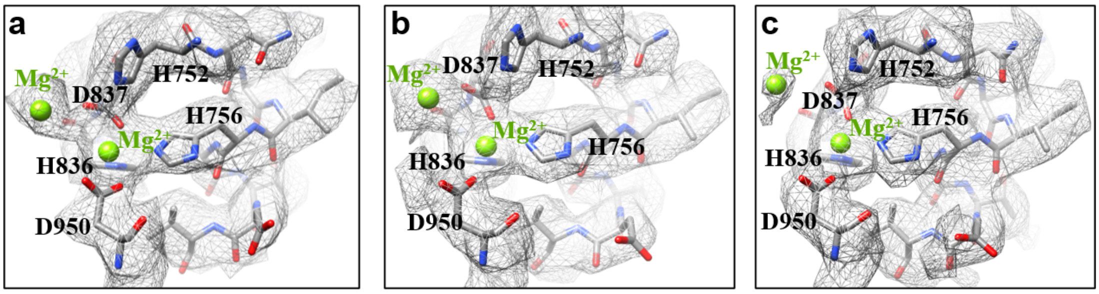

anagrelide DNMDP nauclefine

Supplementary Figure 6. Cryo-EM Densities of Mg^{2+} binding sites. Cryo-EM Densities of Mg^{2+} binding sites of anagrelide- (a), DNMDP- (b) and nauclefine- (c) PDE3A. All these densities were shown with a contour level of mean+30σ.

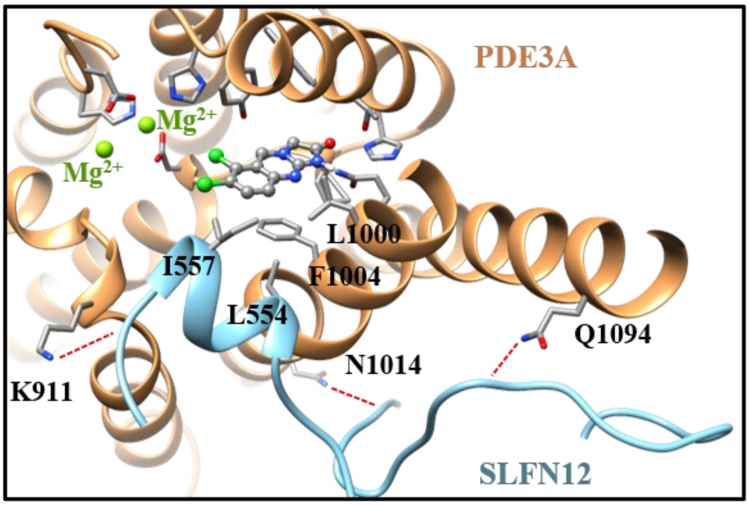

Supplementary Figure 7. The interaction interface between PDE3A (sandy brown) and SLFN12 (light blue). The interaction interface is composed of the strong hydrophobic interaction mediated by the anagrelide compound and three dispersed hydrogen bonds indicated by red dotted lines.

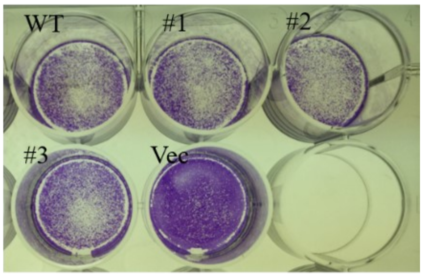

WT : SLFN12 aa1-578
#1 : SLFN12 aa1-550
#2 : SLFN12 aa1-560
#3 : SLFN12 aa1-570

Supplementary Figure 8. The anagrelide-induced PDE3A-SLFN12 interaction depends on SLFN12's C-terminal region. The vehicle, full-length SLFN12(WT), and truncation variants, including residues 1-550 (#1), 1-560 (#2), and 1-570 (#3) fusion with a Flag tag at C-terminus of SLFN12, were inducibly expressed in HeLa (SLFN12$^{-/-}$) cells. The cells were cultured for 72 hs and then cells were stained by methylene blue. This is a representative result from three independent experiments

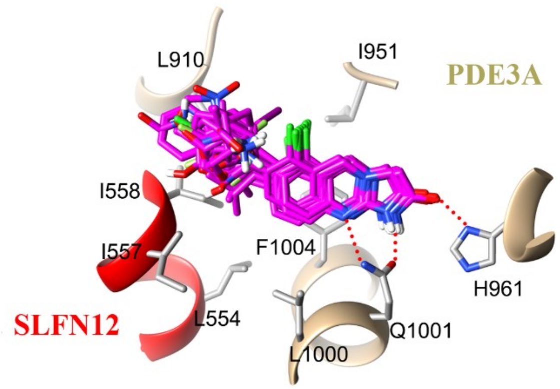

Supplementary Figure 9. The overlapped structures of 7-substituted anagrelide analogs.

Supplementary Tables

Supplementary Table 1. Data collection, reconstruction and modeling information

	SLFN12-PDE3A-anagrelide	SLFN12-PDE3A-DNMDP	SLFN12-PDE3A-nauclefine
Data collection			
Particles	82,930	203,914	60,683
Pixel size (Å)	1.0742	1.091	1.0825
Defocus range (μm)	1-1.8	0.8-2	0.9-1.8
Voltage (kV)	300	300	300
Camera	Gatan K3 Summit	Gatan K3 Summit	Gatan K3 Summit
Electron dose ($e^{-}Å^{-2}$)	50	50	50
Refinement			
Software	Relion3.1	Relion3.1	Relion3.1
Resolution (Å)	3.4	3.2	3.2
Accuracy of rotation (°)	1.83	1.69	1.67
Accuracy of translation (Å)	0.748	0.608	0.704
Map-sharpening *B* factor ($Å^2$)	-158	-138	-133
Model composition			
Chains	4	4	4
Atoms	15266	15304	15290
Protein residues	1878	1878	1878
RNA nucleotides	0	0	0
r.m.s. deviations			
Bonds (Å)	0.009	0.006	0.008
Angles (°)	0.835	0.684	0.730
Ramachandran plot			
Favored (%)	85.93	87.97	87.16
Allowed (%)	13.96	11.92	12.84
Outliers (%)	0.11	0.11	0
Clash score	7.66	5.29	5.89
MolProbity score	2.06	1.88	1.94
Rotamer outliers (%)	0.12	0.36	0.12

Supplementary Table 2. Structure-activity relationship study of anagrelide analogues

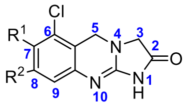

Compounds	R^1	R^2	IC_{50}(nM)	Log P	CLogP	Compounds	R^1	R^2	IC_{50}(nM)	Log P	CLogP
Anagrelide	Cl	H	6.67	2.08	1.02	A12	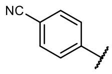	H	2.38	3.23	1.50
A1	Br	H	2.53	2.36	1.15	A13	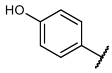	H	12.30	2.81	1.46
A2	F	H	34.19	1.68	0.57	A14	H	H	42.88	1.53	0.43
A3	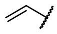	H	10.40	2.16	1.15	A15	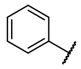	Cl	1.14	3.76	2.53
A4	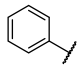	H	0.56	3.20	2.07	A16	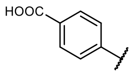	H	ND	2.76	1.83
A5	Cl	Cl	0.70	2.64	1.62	A17	Br	Cl	1.30	2.91	1.73
A6	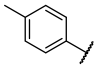	H	0.30	3.69	2.57	A18	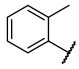	H	1.09	3.69	2.27
A7	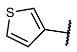	H	1.77	3.13	1.72	A19	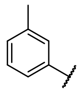	H	1.37	3.69	2.57
A8	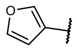	H	0.60	1.76	1.24	A20	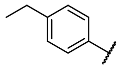	H	1.80	4.11	3.10
A9	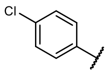	H	1.81	3.76	2.78	A21	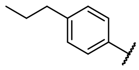	H	ND	4.52	3.62
A10	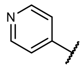	H	6.22	1.86	0.59	A22	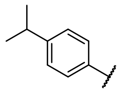	H	ND	4.44	3.49
A11	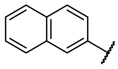	H	ND[a]	4.2	3.24						

[a] Not detected

Supplementary Table 3. Newly generated ligands and MM-GB/SA scoring

Ligands	Chemical Structures	smiles	MM-GB/SA scoring (kcal/mol)
1	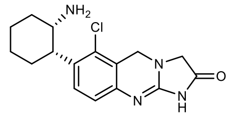	c1c2CN3CC(=O)NC3=Nc2ccc1[<br>C@H]1[C@H](CCCC1)N	-46.58
2	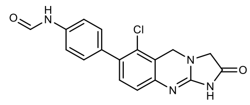	Clc1c2CN3CC(=O)NC3=Nc2ccc<br>1c1ccc(cc1)NC=O	-40.38
3	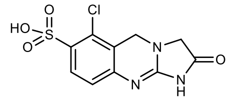	Clc1c2CN3CC(=O)NC3=Nc2ccc<br>1S(=O)(=O)O	-38.11
4	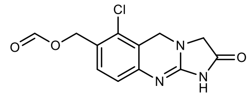	Clc1c2CN3CC(=O)NC3=Nc2ccc<br>1COC=O	-38.09
5	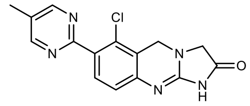	Clc1c2CN3CC(=O)NC3=Nc2ccc<br>1c1ncc(cn1)C	-37.42
6	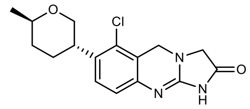	Clc1c2CN3CC(=O)NC3=Nc2ccc<br>1[C@@H]1CC[C@H](OC1)C	-36.07
7	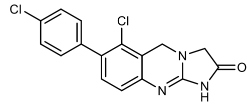	Clc1c2CN3CC(=O)NC3=Nc2ccc<br>1c1ccc(cc1)Cl	-35.19
8	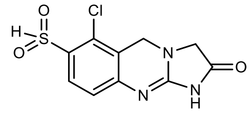	Clc1c2CN3CC(=O)NC3=Nc2ccc<br>1S(=O)=O	-34.45
9	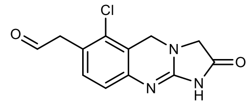	Clc1c2CN3CC(=O)NC3=Nc2ccc<br>1CC=O	-34.04
10	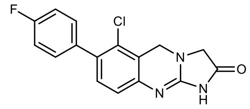	Clc1c2CN3CC(=O)NC3=Nc2ccc<br>1c1ccc(cc1)F	-33.89
11	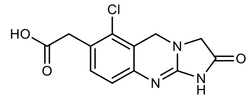	Clc1c2CN3CC(=O)NC3=Nc2ccc<br>1CC(=O)O	-33.64
12	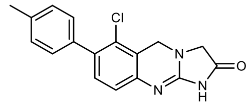	Clc1c2CN3CC(=O)NC3=Nc2ccc<br>1c1ccc(cc1)C	-33.46
13	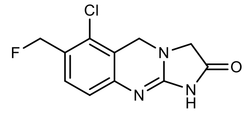	Clc1c2CN3CC(=O)NC3=Nc2ccc<br>1CF	-32.29
14	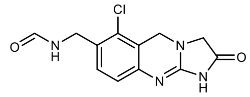	Clc1c2CN3CC(=O)NC3=Nc2ccc<br>1CNC=O	-31.94
15	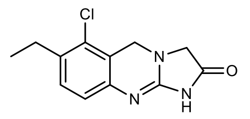	Clc1c2CN3CC(=O)NC3=Nc2ccc<br>1CC	-31.86

16	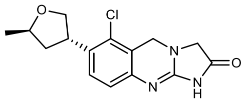	Clc1c2CN3CC(=O)NC3=Nc2ccc1[C@H]1CO[C@@H](C1)C	-31.83
17	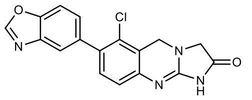	Clc1c2CN3CC(=O)NC3=Nc2ccc1c1ccc2ocnc2c1	-31.78
18	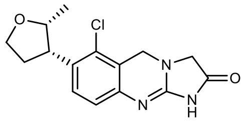	Clc1c2CN3CC(=O)NC3=Nc2ccc1[C@@H]1CCO[C@@H]1C	-30.91
19	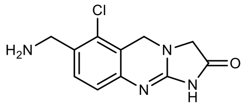	Clc1c2CN3CC(=O)NC3=Nc2ccc1CN	-30.87
20	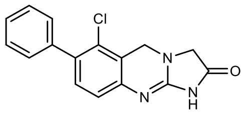	Clc1c2CN3CC(=O)NC3=Nc2ccc1c1ccccc1	-30.83
21	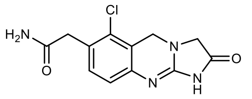	Clc1c2CN3CC(=O)NC3=Nc2ccc1CC(=O)N	-30.52
22	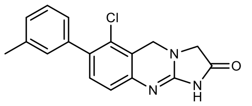	Clc1c2CN3CC(=O)NC3=Nc2ccc1c1cc(ccc1)C	-30.27
23	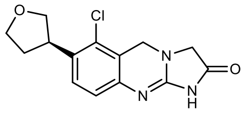	Clc1c2CN3CC(=O)NC3=Nc2ccc1[C@H]1COCC1	-30.19
24	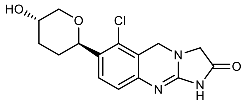	Clc1c2CN3CC(=O)NC3=Nc2ccc1[C@H]1CC[C@@H](CO1)O	-29.80
25	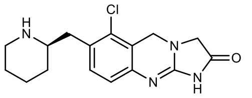	Clc1c2CN3CC(=O)NC3=Nc2ccc1C[C@H]1CCCC[NH2]1	-29.13
26	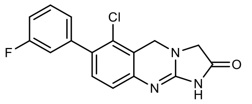	Clc1c2CN3CC(=O)NC3=Nc2ccc1c1cccc(c1)F	-28.93
27	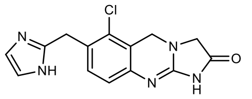	Clc1c2CN3CC(=O)NC3=Nc2ccc1Cc1[nH]ccn1	-28.74
28	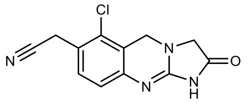	Clc1c2CN3CC(=O)NC3=Nc2ccc1CC#N	-28.67
29	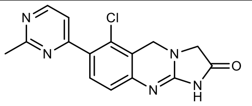	Clc1c2CN3CC(=O)NC3=Nc2ccc1c1ccnc(n1)C	-28.29
30	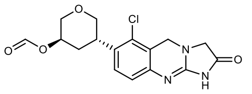	Clc1c2CN3CC(=O)NC3=Nc2ccc1[C@@H]1C[C@H](COC1)OC=O	-28.05
31	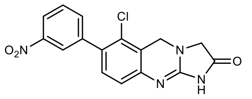	Clc1c2CN3CC(=O)NC3=Nc2ccc1c1cccc(c1)N(=O)=O	-28.04
32	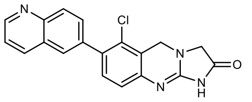	Clc1c2CN3CC(=O)NC3=Nc2ccc1c1ccc2c(c1)cccn2	-27.71

33	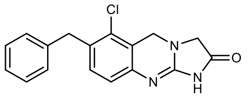	Clc1c2CN3CC(=O)NC3=Nc2ccc1Cc1ccccc1	-27.60
34	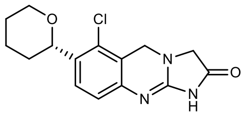	Clc1c2CN3CC(=O)NC3=Nc2ccc1[C@@H]1CCCCO1	-26.81
35	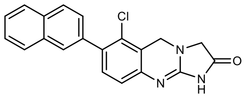	Clc1c2CN3CC(=O)NC3=Nc2ccc1c1cc2c(cccc2)cc1	-25.86
36	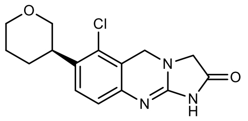	Clc1c2CN3CC(=O)NC3=Nc2ccc1[C@H]1CCCOC1	-25.22
37	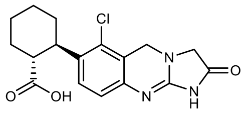	Clc1c2CN3CC(=O)NC3=Nc2ccc1[C@@H]1CCCC[C@H]1C(=O)O	-25.16
38	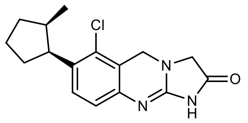	Clc1c2CN3CC(=O)NC3=Nc2ccc1[C@@H]1[C@@H](CCC1)C	-24.82
39	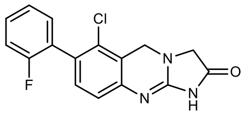	Clc1c2CN3CC(=O)NC3=Nc2ccc1c1c(cccc1)F	-24.73
40	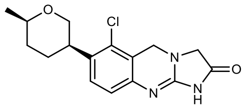	Clc1c2CN3CC(=O)NC3=Nc2ccc1[C@H]1CC[C@H](OC1)C	-24.21
41	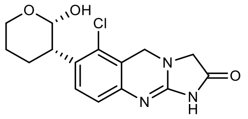	Clc1c2CN3CC(=O)NC3=Nc2ccc1[C@@H]1CCCO[C@@H]1O	-24.07
42	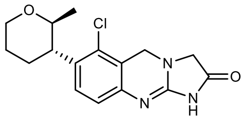	Clc1c2CN3CC(=O)NC3=Nc2ccc1[C@@H]1CCCO[C@H]1C	-23.59
43	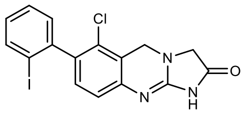	Clc1c2CN3CC(=O)NC3=Nc2ccc1c1ccccc1I	-22.74
44	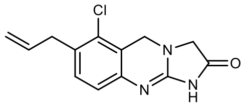	Clc1c2CN3CC(=O)NC3=Nc2ccc1CC=C	-21.18

Supplementary Table 4 List of primers

PDE3A-F (MluI)	CGACGCGT ATGGCAGTGCCCGGCGACGC
PDE3A-R (MluI)	CGACGCGT TCACTGGTCTGGCTTTTGGG
PDE3A(613-1108)-F(BamHI)	CGCGGATCCATGAGTAGAACAGATGACACTG
PDE3A(613-1108)-R(MluI)	CGACGCGT AGGGGTCTGGTCCAGGGATT
PDE3A(669-1108)-F(BamHI)	CGCGGATCC ATGAAACCAATTCTTGCTCCCGA
PDE3A(608-1141)-F(BamHI)	CGCGGATCC ATGGCCACTCGGACACCAAGTAG
PDE3A(608-1141)-R(MluI)	CGACGCGT CTGGTCTGGCTTTTGGGTTG
PDE3A(661-1141)-F(BamHI)	CGCGGATCC ATGCCTGAGACCATGATGTTTCTG
PDE3A(679-1141)-F(BamHI)	CGCGGATCC ATGGATAACCTGGACTCAAT
PDE3A(Y751A)-F	TATAGGGATATTCCT GCT CATAACAGAATCCAT
PDE3A(Y751A)-R	ATGGATTCTGTTATG AGC AGGAATATCCCTATA
PDE3A(H961A)-F	AAATGTAAAGAACTC GCT CTTCAGTGGACAGAT
PDE3A(H961A)-R	ATCTGTCCACTGAAG AGC GAGTTCTTTACATTT
PDE3A(L1000A)-F	CCTCAGCTGGCCAAC GCT CAGGAATCCTTCATC
PDE3A(L1000A)-R	GATGAAGGATTCCTG AGC GTTGGCCAGCTGAGG
PDE3A(Q1001A)-F	CAGCTGGCCAACCTT GCG GAATCCTTCATCTCT
PDE3A(Q1001A)-R	AGAGATGAAGGATTC CGC AAGGTTGGCCAGCTG
PDE3A(F1004A)-F	AACCTTCAGGAATCC GCC ATCTCTCACATTGTG
PDE3A(F1004A)-R	CACAATGTGAGAGAT GGC GGATTCCTGAAGGTT
SLFN12-F (NotI)	CG GCGGCCGC ATGAACATCAGTGTTGATTTG
SLFN12-R(NotI)	CG GCGGCCGC TCAGGTGAGCCTTCGACAAGA
SLFN12-F(SpeI)	CTAGACTAGT ATGAACATCAGTGTTGATTTG
SLFN12-F(MluI)	CGACGCGT TCAGGTGAGCCTTCGACAAGA
SLFN12(1-550)-F (NotI)	CG GCGGCCGC TCAAAAGGAAAACTGGTCTCTCA
SLFN12(1-560)-R(NotI)	CG GCGGCCGC TCATATACCGATTATCTGGTATA
SLFN12(1-570)-R(NotI)	CG GCGGCCGC TCACATCTTTTTATCATTCTTCT

Supplementary Note 1

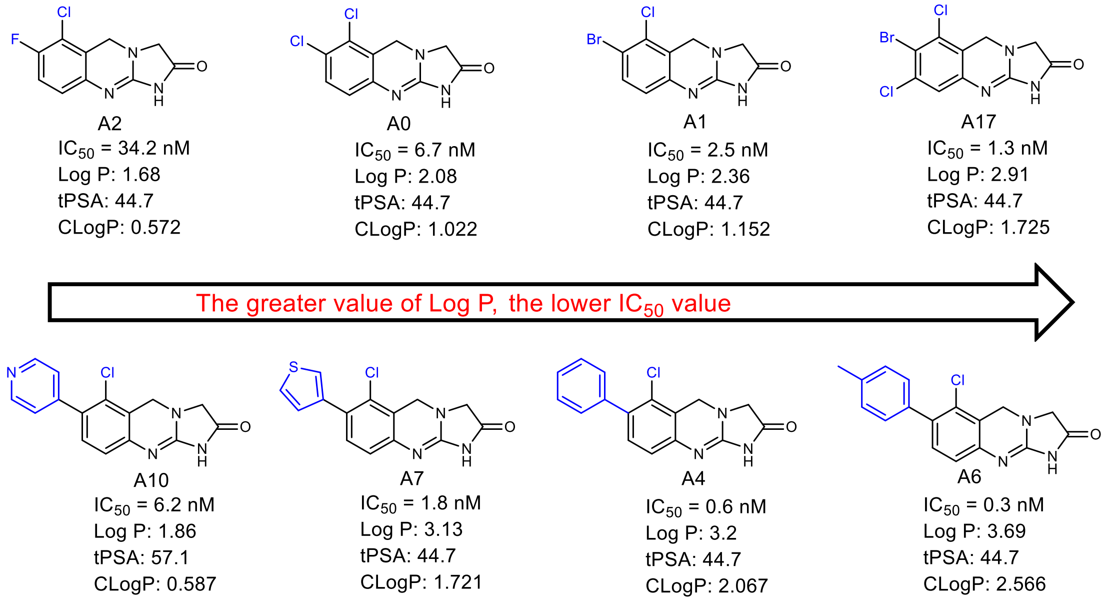

We did not observe the linear correlation of the logP value with IC_{50} of all the molecules Supplementary Table 2. However, as we indicated in the text, the hydrophobic interaction is crucial for the binding site of the PDE3A/SLFN12 interface, we then analyzed the correlation of the logP value and IC_{50} of the molecules with the similar interaction pattern at this interface and indeed observed the same tendency that the greater the value of LogP, the lower IC_{50} value. We admit that the interaction situation in this binding site is much more complicated and there are certainly other parameters that might affect the interaction, such as the steric interruption, π-π-stacking interactions and so on, but likely, the hydrophobic interaction plays a key role for the gluing activity in the current study.

Supplementary Methods

Chemical Synthesis. All reactions were carried out under an atmosphere of nitrogen in flame-dried glassware with magnetic stirring unless otherwise indicated. Commercially obtained reagents were used as received. Solvents were dried by passage through an activated alumina column under argon. Liquids and solutions were transferred via syringe. All reactions were monitored by thin-layer chromatography with E. Merck silica gel 60 F254 pre-coated plates (0.25 mm). 1H and 13C NMR spectra were recorded on Varian Inova-400 or 500 spectrometers. Data for 1H NMR spectra are reported relative to CDCl3 (7.26 ppm), CD_3OD (3.31 ppm), or DMSO-d6 (2.50 ppm) as an internal standard and are reported as follows: chemical shift (δppm), multiplicity (s = singlet, d = doublet, t = triplet, q = quartet, sept = septet, m = multiplet, br = broad), coupling constant *J* (Hz), and integration. Data for 13C NMR spectra are reported relative to $CDCl_3$ (77.23 ppm), CD_3OD (49.00 ppm) or DMSO-d6 (39.52 ppm) as an internal standard and are reported in terms of chemical shift (δ ppm). Samples preparation and purity analysis were conducted on Waters HPLC (Column: XBridge C18, 5μm, 19 x 150 mm) with 2998PDA and 3100MS detectors, and Waters UPLC (Column: BEH C18, 1.7μm, 2.1 x 50 mm) with PDA and SQD MS detectors, using ESI as ionization. HRMS data were obtained on a Thermo Q Exactive.

All new compounds were synthesized as indicated in detail in the following schemes. Compound A2 was synthesized according to the same synthetic route as compound A1 using different starting materials 3-fluoro-2-chlorobenzaldehyde. Di-substituted compounds A3, A4, A6-A14, A16, A18-A22 were synthesized using a Suzuki coupling reaction from A1. Compounds A5 were synthesized according to the same synthetic route as compound A17 using different starting materials 3,4,5-trichloroaniline. Tri-substituted compound A15 was synthesized using a Suzuki coupling reaction from A17. A14 was the by-product of the Suzuki coupling reaction for di-substituted compounds.

Scheme 1. Synthesis of di-substitution compounds

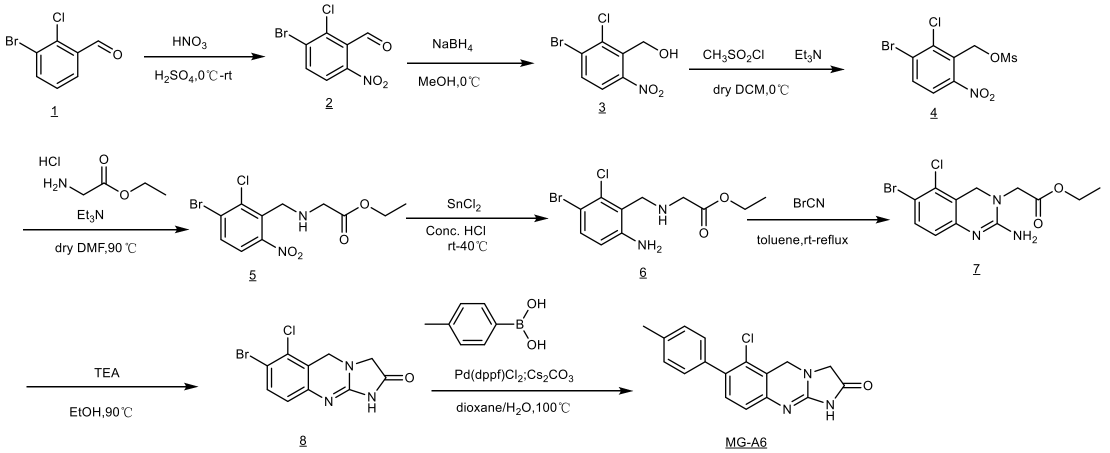

Synthesis of compound 2

In a flame-dried 50 mL 2-necked round-bottom flask, concentrated sulfuric acid (15mL), and 3-bromo-2-chlorobenzaldehyde **(**2g, 9.11mmol, 1.0equiv) was added in small portions while stirring. Conc. nitric acid (3.0mL) was added dropwise at 0°C. The reaction mixture was stirred at 0 ^{o}C for 30min and then warmed to rt overnight. It was checked by TLC until completion. The reaction mixture was poured into 100mL ice water, the mixture was extracted with EA (3 x 50 mL), washed with brine and concentrated. The mixture was purified by flash chromatography on silica gel (PE:EA = 300:1) to give compound **2** as a white solid (1.1g, 46% yield).

Synthesis of compound 3

In a flame-dried 50 mL round-bottom flask, 3-bromo-2-chloro-6-nitrobenzaldehyde (1.1g, 4.18mmol, 1.0equiv)was dissolved in methanol (20.0mL). The reaction mixture was cooled to 0°C. $NaBH_4$ (316mg, 8.36mmol, 2.0equiv) was added in small portions. The reaction mixture was stirred at 0 ^{o}C for 30min. TLC showed the starting material was consumed. The reaction mixture was quenched with ice water (50mL), extracted with EA (3 x 50 mL), washed with brine and concentrated. The mixture was purified by flash chromatography on silica gel (PE:EA = 15:1) a to give compound **3** as a white solid (1.1g, 99% yield).

Synthesis of compound 4

In a flame-dried 50 mL round-bottom flask, (3-bromo-2-chloro-6-nitrophenyl)methanol(1.1g, 4.15mmol, 1.0equiv)was dissolved in dry DCM (11.0mL), then dry Et_3N (0.87mL, 6.23mmol, 1.5equiv) was added. The mixture was cooled to 0°C. Methanesulfonyl chloride (0.38mL, 4.98mmol, 1.2equiv) was added in small portions. The reaction mixture was stirred at 0 °C for 30min. TLC showed the starting material was consumed. The reaction mixture was quenched with ice water (50mL), extracted with EA (3 x 50 mL), washed with Sat. $NaHCO_3$ and brine and then concentrated. The mixture was purified by flash chromatography on silica gel (PE:EA = 10:1) to give compound **4** as a white solid (1.35g, 95% yield).

Synthesis of compound 5

In a flame-dried 50 mL round-bottom flask, 3-bromo-2-chloro-6-nitrobenzyl methanesulfonate (1.35g, 3.94mmol, 1.0equiv), glycine ethyl ester hydrochloride (825mg, 5.91mmol, 1.5equiv), were dissolved in dry DMF (27.0mL), and then dry Et_3N (1.37mL, 9.85mmol, 2.5equiv,) was added. The reaction mixture was stirred at 90°C for 14h. TLC showed the starting material was consumed. The reaction mixture was quenched with ice water (50mL), extracted with EA (3 x 50 mL), washed with brine and concentrated. The mixture was purified by flash chromatography on silica gel (PE:EA = 10:1) to give compound **5** as a white solid (1.15g, 83% yield).

Synthesis of compound 6

In a flame-dried 100 mL 2-necked round-bottom flask, Conc. HCl (23mL), and ethyl 2-((3-bromo-2-chloro-6-nitrobenzyl)amino)acetate (1.15g, 3.27mmol, 1.0equiv) was added in small portions. Stannous chloride (3.1g, 16.35mmol, 5.0equiv) dissolved in Conc. HCl (13mL) was added dropwise at rt. The reaction mixture was stirred at 40 °C for 1h and checked by TLC. The reaction mixture was poured into 100mL ice water (with 35mL $NH_3 \cdot H_2O$), extracted with EA (3 x 50 mL), washed with brine and concentrated. The mixture was purified by flash chromatography on silica gel (PE:EA = 15:1) to give compound **6** as a white solid (913mg, 87% yield).

Synthesis of compound 7

In a flame-dried 50 mL round-bottom flask, ethyl 2-((6-amino-3-bromo-2-chlorobenzyl)amino)acetate (913mg, 2.85mmol, 1.0equiv) was dissolved in dry toluene (20.0mL), and then cyanogen bromide (453mg, 4.28mmol, 1.5equiv) added in small portions while stirring at rt. The reaction mixture was stirred at 110 °C for 14h. It was checked by TLC until completion. The reaction mixture was filtered by sand core funnel to get the crude product **7** as a solid (1.02g).

Synthesis of compound A1

In a flame-dried 50 mL 2-necked round-bottom flask, ethyl 2-(2-amino-6-bromo-5-chloroquinazolin-3(4H)-yl)acetate (1.02g, 2.95mmol, 1.0equiv) and triethylamine (0.61mL, 4.42mmol, 1.5equiv) were dissolved in ethanol (15.0mL) at rt. The reaction mixture was stirred at 100 °C for 14h. It was checked by TLC until completion. The reaction mixture was filtered by sand core funnel to get the crude product **A1** as a light yellow solid (729mg).

Synthesis of compounds A3, A4, A6-A14, A16, A18-A22

In a flame-dried 25 mL tube, **A1** (100mg, 0.334mmol, 1.0equiv), boronic acid (0.40mmol, 1.2equiv), Cs_2CO_3 (436mg, 1.34mmol, 4.0equiv), and Pd(dppf)Cl_2 (36.6mg, 0.05mmol, 0.15equiv) were dissolved in dioxane/H_2O (4:1, 10mL). The mixture was degassed and filled with argon. The reaction mixture was stirred at 100 °C for 5h. It was checked by TLC until completion. The reaction mixture was purified by flash chromatography on silica gel (DCM:MeOH = 60:1) to give the product **A3, A4, A6-A14, A16, A18-A22**.

Scheme 2. Synthesis of tri-substituted compounds

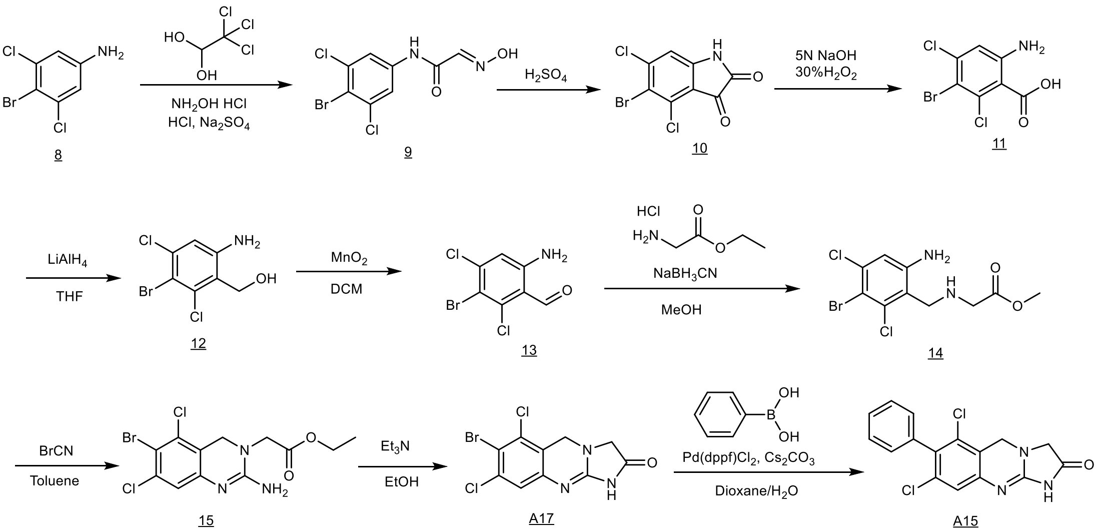

Synthesis of compound 9

A flame-dried 250 mL round-bottom flask 4-bromo-3,5-dichloroaniline (1.0g, 5.13mmol, 1.0equiv) was dissolved in 70.0mL H_2O, and then chloral hydrate (1.7g, 9.09mmol, 1.8equiv), hydroxylamine hydrochloride (2.0g, 8.57mmol, 5.6equiv), crystallized sodium sulfate (2.5g, 17.60mmol, 3.2equiv), and concentrated hydrochloric acid (0.8mL) were added. The reaction mixture was stirred at 80 °C for 4h. The reaction mixture was cooled to 0 °C and the sediments filtered to get the crude product **9** (1.36g, 85%yield) was used directly without further purification.

Synthesis of compound 10

A flame-dried 50 mL round-bottom flask, compound **9** (1.36g, 4.36mmol, 1.0equiv) and concentrated sulfuric acid (10.0mL) was added. The reaction mixture was stirred at 80 °C for 1h. Then the reaction mixture was cooled to room temperature and poured upon ten to twelve times its volume of cracked ice. The mixture was filtered, washed 5 times with cold water to remove the sulfuric acid, and then dried. The crude reaction mixture was purified by flash chromatography on silica gel (PE:EA = 15:1) to give the compound **10** as a brown solid (0.93g, 72% yield).

Synthesis of compound 11

A flame-dried 50 mL round-bottom flask, compound **10** (0.93g, 3.15mmol, 1.0equiv), 5 M aqueous sodium hydroxide solution (3.0 mL) were added, and then hydrogen peroxide (30%) solution (1.2 mL) was dropwise over 5min. The reaction mixture was stirred at room temperature for 4h. The concentrated hydrochloric acid was added to adjust the pH to 5-6. The reaction mixture extracted with EA (3 x 100 mL), washed with brine and then concentrated to give the crude product **11** (870mg) was used directly without further purification.

Synthesis of compound 12

A flame-dried 25 mL round-bottom, compound **11** (770mg, 2.71mmol, 1.0equiv) was dissolved in tetrahydrofuran (10 mL), and then lithium aluminum hydride (141mg, 3.71mmol, 1.4equiv) in 20 mL tetrahydrofuran was added in portion at 0 °C. The reaction mixture was stirred at 0 °C for 30min before it was gradually warmed to rt overnight. The reaction mixture was poured into 10 mL ice saturated sodium bicarbonate solution, extracted with EA (3 x 100 mL) washed with brine and

then concentrated. The mixture was purified by flash chromatography on silica gel (PE:EA = 10:1) to give compound **12** as a light yellow solid (510mg, 70%yield).

Synthesis of compound 13

A flame-dried 25 mL round-bottom flask, compound **12** (40mg, 0.15mmol, 1.0equiv) was dissolved in dichloromethane (5 mL), and then manganese oxide (80mg, 0.92mmol, 6.1equiv) was added. The reaction mixture was stirred at rt overnight. The reaction mixture was filtered by diatomaceous earth with suction and washed by dichloromethane. The crude mixture was purified by flash chromatography on silica gel to give the compound **13** as a light yellow solid (36mg, 90%yield).

Synthesis of compound 14

A flame-dried 25 mL round-bottom flask, compound **13** (64mg, 0.24mmol, 1.0equiv) was dissolved in methanol (7 mL), glycine ethyl ester hydrochloride (40mg, 0.28mmol, 1.2equiv) and sodium cyanoborohydride (18mg, 0.28mmol, 1.2equiv) was added. The reaction mixture was stirred at 35 ^{o}C for overnight. The reaction mixture was poured into 10 ml ice saturated sodium bicarbonate solution, extracted with EA (3 x 300 mL) washed with brine and then concentrated. The crude reaction mixture was purified by flash chromatography on silica gel (PE:EA = 5:1) to give the compound **14** as a white solid (47mg, 57%yield).

Synthesis of compound 15

A flame-dried 25 mL round-bottom flask, compound **14 (**47mg, 0.13mmol, 1.0equiv) was dissolved in dry toluene (1.3 mL), and then cyanogen bromide (46mg, 0.40mmol, 3.0equiv) was added in small portions while stirring at rt. The reaction mixture was stirred at rt overnight. The reaction mixture was filtered by sand core funnel (washed with dry toluene) to give the compound **15** as a white solid (36mg, 71% yield).

Synthesis of compound A17

A flame-dried 50 mL 2-necked round-bottom flask, compound **15 (**36mg, 0.09mmol, 1.0equiv) and triethylamine (17μL, 0.12mmol, 1.3equiv) were dissolved in ethanol (1.5 mL) at rt. The reaction mixture was stirred at 80 ^{o}C for 2.5 h. The reaction mixture was filtered by sand core funnel (washed by ethanol) to give the product **A17** as a light yellow solid (12mg, 38% yield).

Synthesis of compound A15

A flame-dried 10 mL sealed tube, **A17** (5.5mg, 0.016 mmol, 1.0 equiv) , phenylboronic acid (2.4mg, 0.019mmol, 1.2equiv), caesium carbonate (21.4mg, 0.066mmol, 4.0equiv), Pd(dppf)Cl2 (1.8mg, 0.002mmol, 0.15equiv) were dissolved in dioxane/H_2O (4:1, 1.0mL), sealed, degassed and filled with argon. The reaction mixture was heated to 100 ^{o}C for 2 h. It was checked by UPLC until completion. The reaction mixture was purified by flash chromatography on silica gel (DCM:MeOH = 60:1) to give the product **A15** as a white solid (2.3mg, 42%yield).

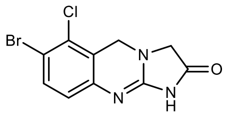

A1

^{1}H NMR (400 MHz, DMSO-d_6): δ 3.87 (s, *2*H), 4.58 (s, 2H), 6.86 (d, *J* = 6.3 Hz, 1H), 7.62 (d, 1H); HRMS (ESI) calculated for [M+H]$^+$ 299.9539, found 299.9531.

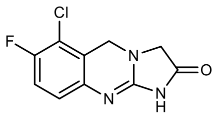

A2

^{1}H NMR (400 MHz, DMSO-d_6): δ 3.87 (s, 2H), 4.52 (s, 2H), 6.85 (dd, *J* = 8.9Hz, 1H), 7.27 (t, *J* = 9.0 Hz, 1H), 11.13 (s, 1H); HRMS (ESI) calculated for [M+H]$^+$ 240.0340, found 240.0334.

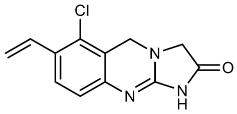

A3

^{1}H NMR (400 MHz, MeOD + $CDCl_3$): δ 3.61 (s, *2*H), 4.02 (s, 2H), 5.34 (d, *J* = 10.4 Hz, 1H), 5.71 (d, *J* = 17.4 Hz, 1H), 6.92-6.87 (m, 1H), 7.026.98 (m, 1H), 7.51-7.50 (m, 1H); HRMS (ESI) calculated for [M+H]$^+$ 248.0591, found 248.0582.

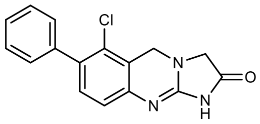

A4

^{1}H NMR (400 MHz, MeOD + $CDCl_3$): δ 4.02 (s, 2H), 4.72 (s, 2H), 6.98 (d, *J* = 8.2 Hz, 1H), 7.25 (d, *J* = 8.2 Hz,, 1H), 7.41-7.34 (m, 5H); HRMS (ESI) calculated for [M+H]$^+$ 298.0747, found 298.0739.

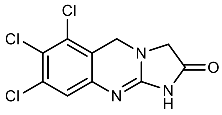

A5

^{1}H NMR (400 MHz, DMSO-d_6): δ 3.88 (s, *2*H), 4.52 (s, 2H), 7.08 (s, 1H); HRMS (ESI) calculated for [M+H]$^+$ 289.9655, found 289.9661.

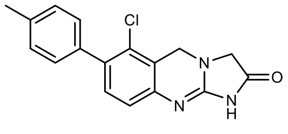

A6

^{1}H NMR (400 MHz, DMSO-d_6): δ 2.37 (s, 3H), 3.87 (s, *2*H), 4.58 (s, 2H), 6.96 (d, *J* = 6.3 Hz, 1H), 7.27-7.24 (m, 5H); HRMS (ESI) calculated for [M+H]$^+$ 312.0903, found 312.0894.

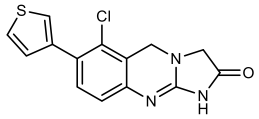

A7

^{1}H NMR (400 MHz, DMSO-d_6): δ 3.74 (s, 2H), 4.51 (s, 2H), 6.86 (d, *J* = 6.0 Hz, 1H), 7.30-7.26 (m, 2H), 7.60-7.58 (m, 2H); HRMS (ESI) calculated for $[M+H]^+$ 304.0311, found 304.0301.

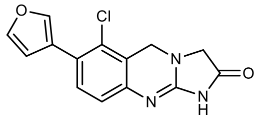

A8

^{1}H NMR (400 MHz, DMSO-d_6): δ 3.87 (s, 2H), 4.58 (s, 2H), 6.83 (dd, *J* = 1.9, 0.9 Hz, 1H), 6.93 (d, *J* = 6.3 Hz, 1H), 7.43 (d, *J* = 6.3 Hz, 1H), 7.75 (t, *J* = 1.7 Hz, 1H), 8.05 (dd, *J* = 1.6, 0.9 Hz, 1H); HRMS (ESI) calculated for $[M+H]^+$ 288.0539, found 288.0531.

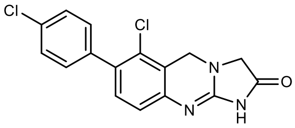

A9

^{1}H NMR (400 MHz, DMSO-d_6): δ 3.87 (s, 2H), 4.58 (s, 2H), 6.97 (d, *J* = 6.3 Hz, 1H), 7.27 (d, *J* = 6.3 Hz, 1H), 7.42-7.38 (m, 2H), 7.52-7.48 (m, 2H); HRMS (ESI) calculated for $[M+H]^+$ 332.0357, found 332.0346.

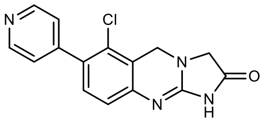

A10

^{1}H NMR (400 MHz, DMSO-d_6): δ 3.87 (s, 2H), 4.58 (s, 2H), 6.98 (d, *J* = 6.3 Hz, 1H), 7.31 (d, *J* = 6.3 Hz, 1H), 7.42 (dd, *J* = 6.0, 2.7 Hz, 2H), 8.63 (dd, *J* = 6.0, 2.7 Hz, 2H); HRMS (ESI) calculated for $[M+H]^+$ 299.0696, found 299.0692.

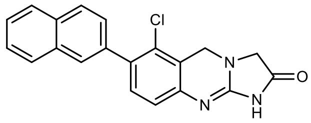

A11

^{1}H NMR (400 MHz, DMSO-d_6): δ 3.87 (s, 2H), 4.61 (s, 2H), 7.00 (d, *J* = 6.3 Hz, 1H), 7.37 (d, *J* = 6.3 Hz, 1H), 7.56-7.52 (m, 3H), 7.97-7.91 (m, 4H); HRMS (ESI) calculated for $[M+H]^+$ 348.0903, found 348.0891.

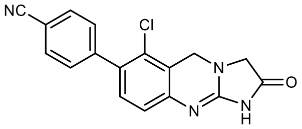

A12

^{1}H NMR (400 MHz, DMSO-d_6): δ 3.84 (s, 2H), 4.57 (s, 2H), 6.96 (d, *J* = 6.3 Hz, 1H), 7.28 (d, *J* =

6.3 Hz, 1H), 7.60-7.58 (m, 2H), 7.92-7.90 (m, 2H); HRMS (ESI) calculated for $[M+H]^+$ 323.0699, found 323.0690.

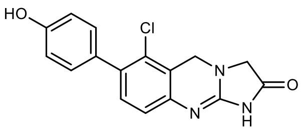

A13

^{1}H NMR (400 MHz, DMSO-d_6): δ 3.86 (s, 2H), 4.57 (s, 2H), 6.81-6.79 (m, 2H), 6.93 (d, *J* = 6.3 Hz, 1H), 7.21-7.16 (m, 3H), 9.57 (s, 1H); HRMS (ESI) calculated for $[M+H]^+$ 314.0696, found 314.0687.

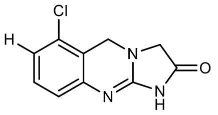

A14

^{1}H NMR (400 MHz, DMSO-d_6): δ 3.84 (s, 2H), 4.52 (s, 2H), 6.89 (dd, *J* = 8.0, 1.1 Hz, 1H), 7.09 (dd, *J* = 8.0, 1.1 Hz, 1H), 7.23 (t, *J* = 8.0 Hz, 1H); HRMS (ESI) calculated for $[M+H]^+$ 222.0434, found 222.0426.

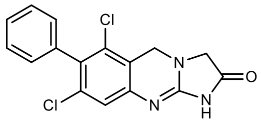

A15

^{1}H NMR (400 MHz, DMSO-d_6): δ 3.81 (s, 2H), 4.48 (s, 2H), 6.97 (s, 1H), 7.22-7.19 (m, 2H), 7.47-7.37 (m, 3H); HRMS (ESI) calculated for $[M+H]^+$ 332.0357, found 332.0346.

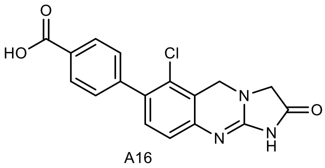

A16

^{1}H NMR (400 MHz, DMSO-d_6): δ 3.65 (s, 2H), 4.47 (s, 2H), 6.83-6.80 (m, 1H), 7.15-7.13 (m, 1H), 7.21 (d, *J* = 12 Hz, 2H), 7.83 (d, *J* = 8.0 Hz, 2H); HRMS (ESI) calculated for $[M+H]^+$ 342.0645, found 342.0636.

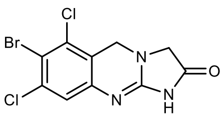

A17

^{1}H NMR (400 MHz, DMSO-d_6): δ 3.90 (s, 2H), 4.52 (s, 2H), 7.05 (s, 1H); HRMS (ESI) calculated for $[M+H]^+$ 333.9149, found 333.9139.

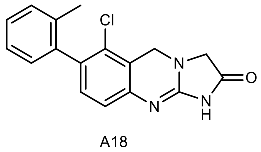

A18

^1H NMR (400 MHz, DMSO-d_6): δ 2.04 (s, 3H), 3.87 (s, *2*H), 4.58 (d, *J* = 4.8 Hz, 2H), 6.97 (d, *J* = 6.3 Hz, 1H), 7.07 (d, *J* = 5.4 Hz, 1H), 7.15 (d, *J* = 6.3 Hz, 1H), 7.26-7.21 (m, 1H), 7.30-7.28 (m, 2H); HRMS (ESI) calculated for $[M+H]^+$ 312.0903, found 312.0894.

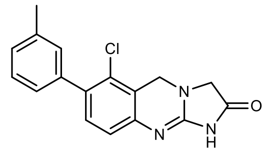

A19

^1H NMR (400 MHz, DMSO-d_6): δ 2.34 (s, 3H), 3.86 (s, 2H), 4.58 (s, 2H), 6.95 (d, *J* = 6.0 Hz, 1H), 7.19-7.15 (m, 3H), 7.24 (d, *J* = 6.0 Hz, 1H), 7.34-7.30 (m, 1H); HRMS (ESI) calculated for $[M+H]^+$ 312.0903, found 312.0894.

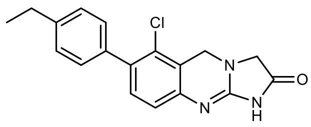

A20

^1H NMR (400 MHz, DMSO-d_6): δ 1.21 (t, *J* = 5.4 Hz, 1H), 2.64 (q, *J* = 5.4 Hz, 2H), 3.87 (s, 2H), 4.58 (s, 2H), 6.96 (d, *J* = 6.3 Hz, 1H), 7.28-7.24 (m, 3H), 7.56-7.48 (m, 2H); HRMS (ESI) calculated for $[M+H]^+$ 326.1060, found 326.1051.

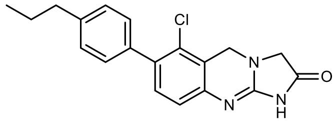

A21

^1H NMR (400 MHz, DMSO-d_6): δ 0.91 (t, *J* = 5.4 Hz, 1H), 1.61 (m, 2H), 2.58 (t, *J* = 5.7 Hz, 2H), 3.87 (s, *2*H), 4.58 (s, *2*H), 6.96 (d, *J* = 6.3 Hz, 1H), 7.30-7.24 (m, 5H); HRMS (ESI) calculated for $[M+H]^+$ 340.1217, found 340.1207.

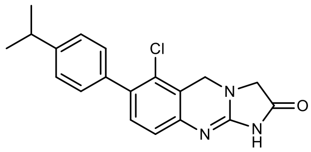

A22

^1H NMR (400 MHz, DMSO-d_6): δ 1.22 (d, *J* = 5.1 Hz, 6H), 2.95-2.89 (m, 1H), 3.87 (s, 2H), 4.58 (s, 2H), 6.96 (d, *J* = 6.3 Hz, 1H), 7.34-7.25 (m, 5H); HRMS (ESI) calculated for $[M+H]^+$ 340.1217, found 340.1207.

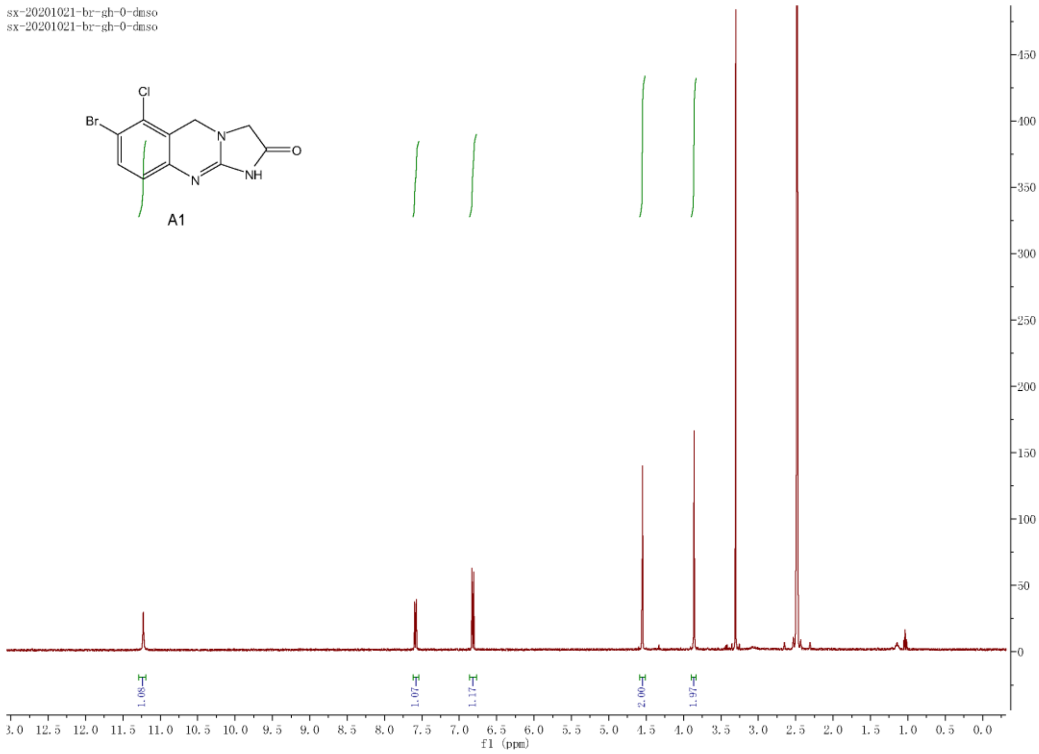
sx-20201021-br-gh-0-dmso
sx-20201021-br-gh-0-dmso
A1
f1 (ppm)

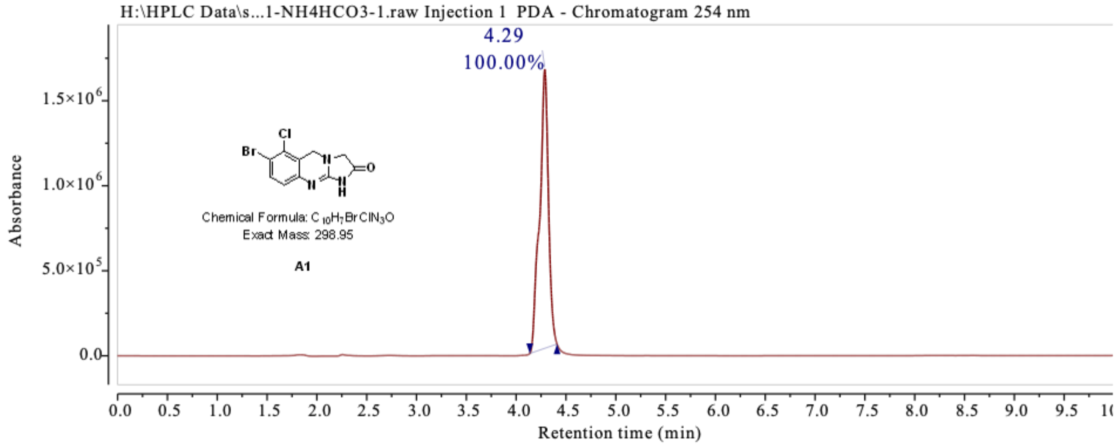
H:\HPLC Data\s...1-NH4HCO3-1.raw Injection 1 PDA - Chromatogram 254 nm
4.29
100.00%
Chemical Formula: C10H7BrClN3O
Exact Mass: 298.95
A1
Absorbance
Retention time (min)

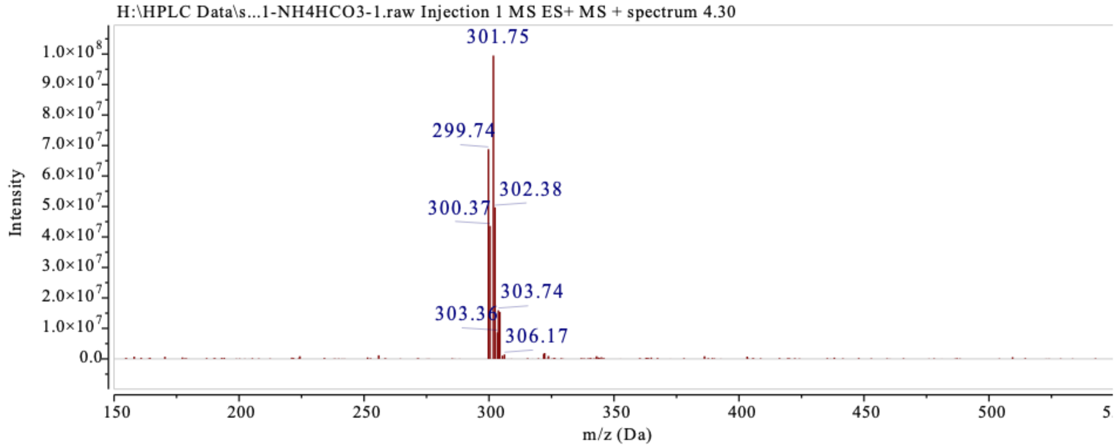
H:\HPLC Data\s...1-NH4HCO3-1.raw Injection 1 MS ES+ MS + spectrum 4.30
301.75
299.74
302.38
300.37
303.74
303.36
306.17
Intensity
m/z (Da)

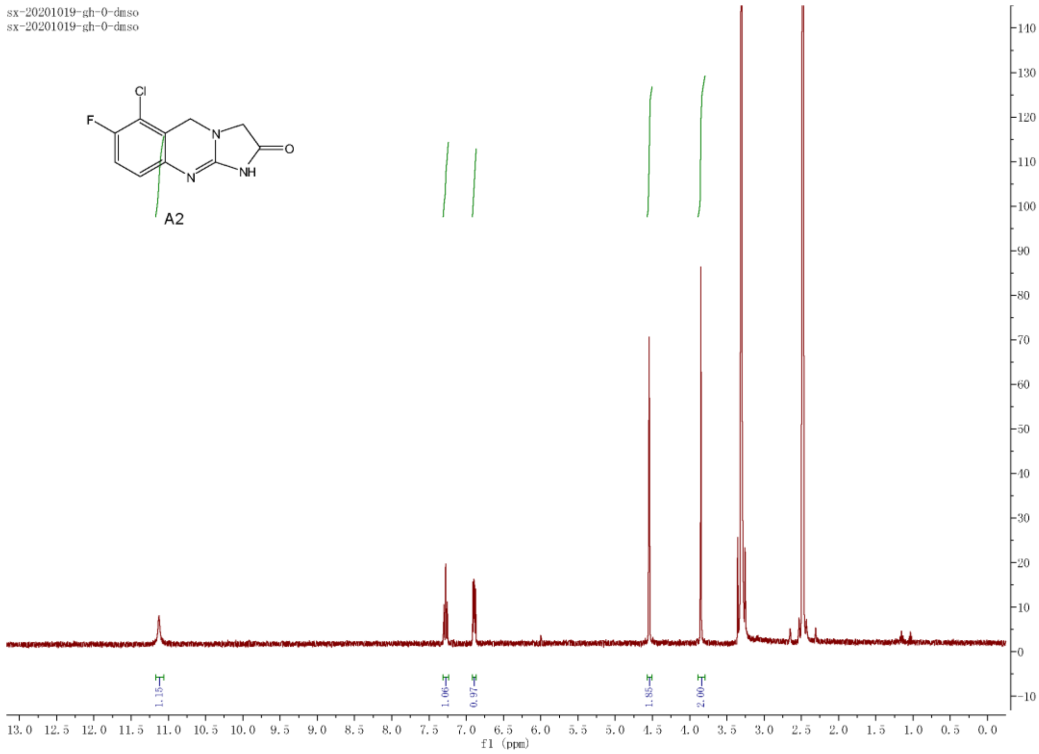
sx-20201019-gh-0-dmso
sx-20201019-gh-0-dmso
Cl
F
N
NH
N
O
A2
1.15
1.06
0.97
1.85
2.00
f1 (ppm)

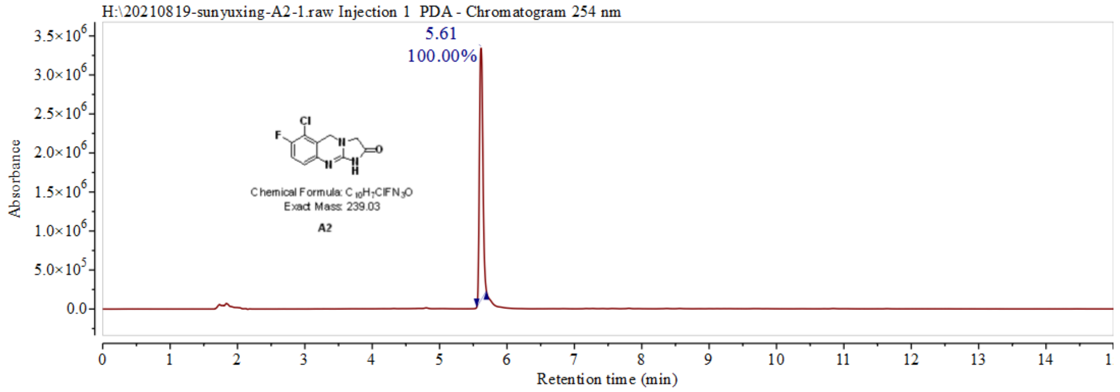
H:\20210819-sunyuxing-A2-1.raw Injection 1 PDA - Chromatogram 254 nm
5.61
100.00%
Chemical Formula: C10H7ClFN3O
Exact Mass: 239.03
A2
Absorbance
Retention time (min)

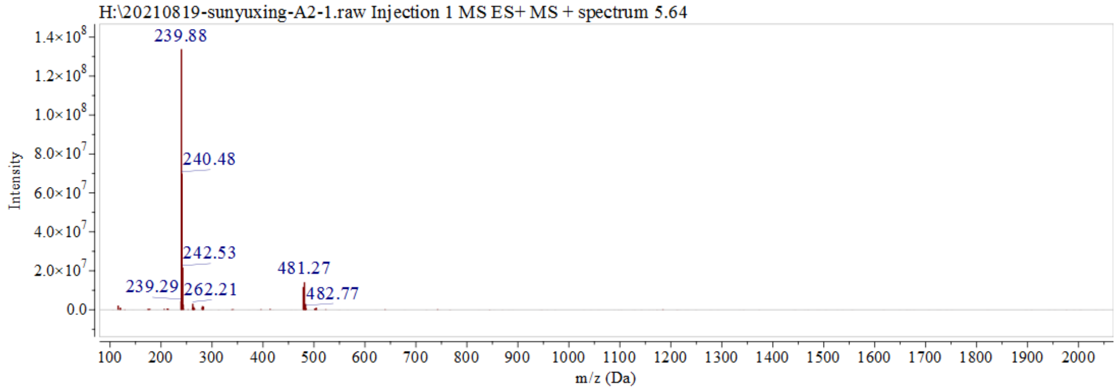
H:\20210819-sunyuxing-A2-1.raw Injection 1 MS ES+ MS + spectrum 5.64
239.88
240.48
242.53
239.29
262.21
481.27
482.77
Intensity
m/z (Da)

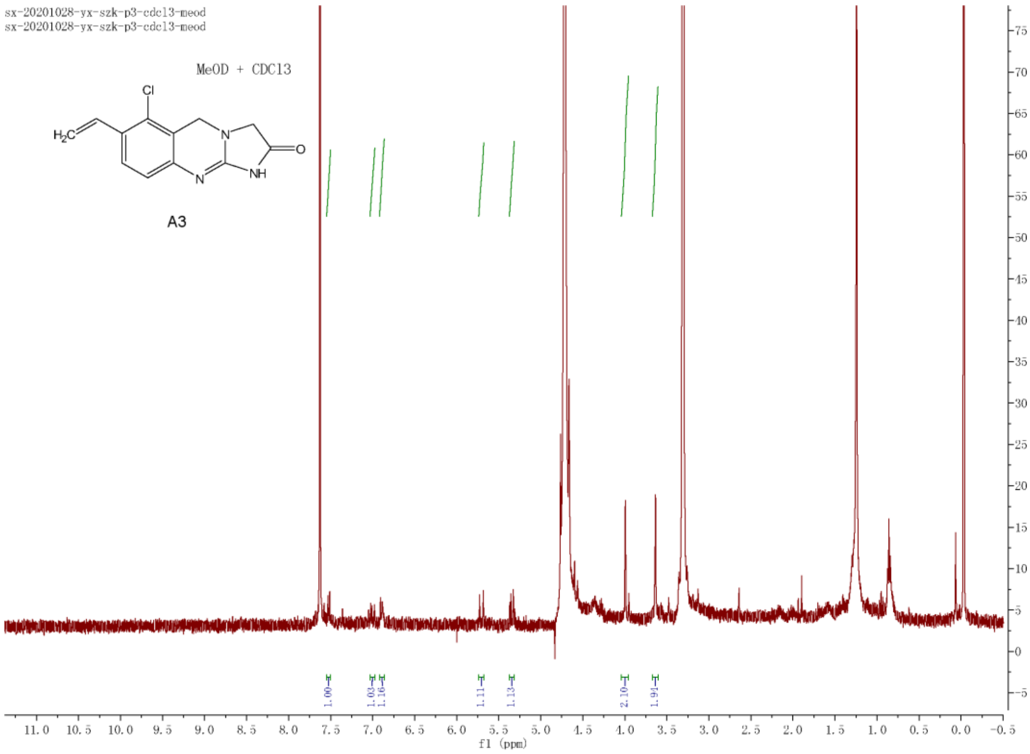
sx-20201028-yx-szk-p3-cdcl3-meod
sx-20201028-yx-szk-p3-cdcl3-meod
MeOD + CDCl3
A3
f1 (ppm)

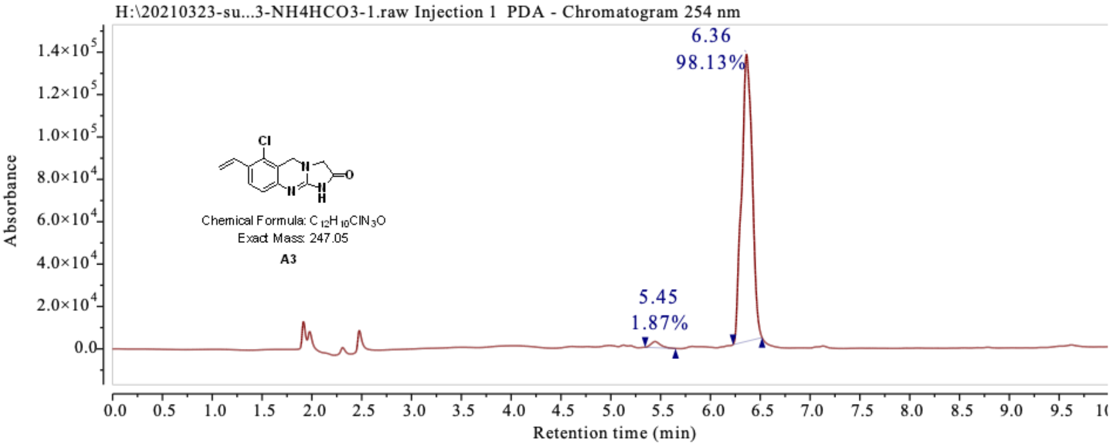
H:\20210323-su...3-NH4HCO3-1.raw Injection 1 PDA - Chromatogram 254 nm
6.36
98.13%
5.45
1.87%
Chemical Formula: C12H10ClN3O
Exact Mass: 247.05
A3
Absorbance
Retention time (min)

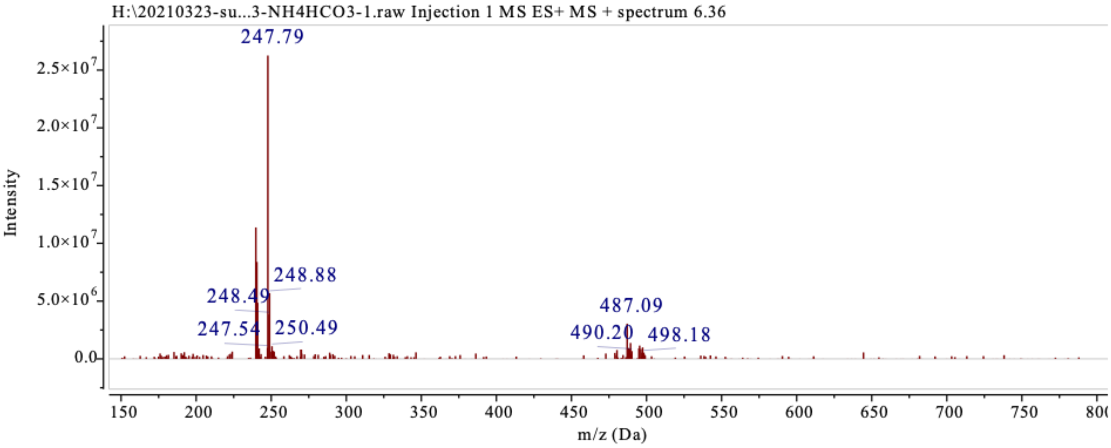
H:\20210323-su...3-NH4HCO3-1.raw Injection 1 MS ES+ MS + spectrum 6.36
247.79
248.88
248.49
247.54
250.49
487.09
490.20
498.18
Intensity
m/z (Da)

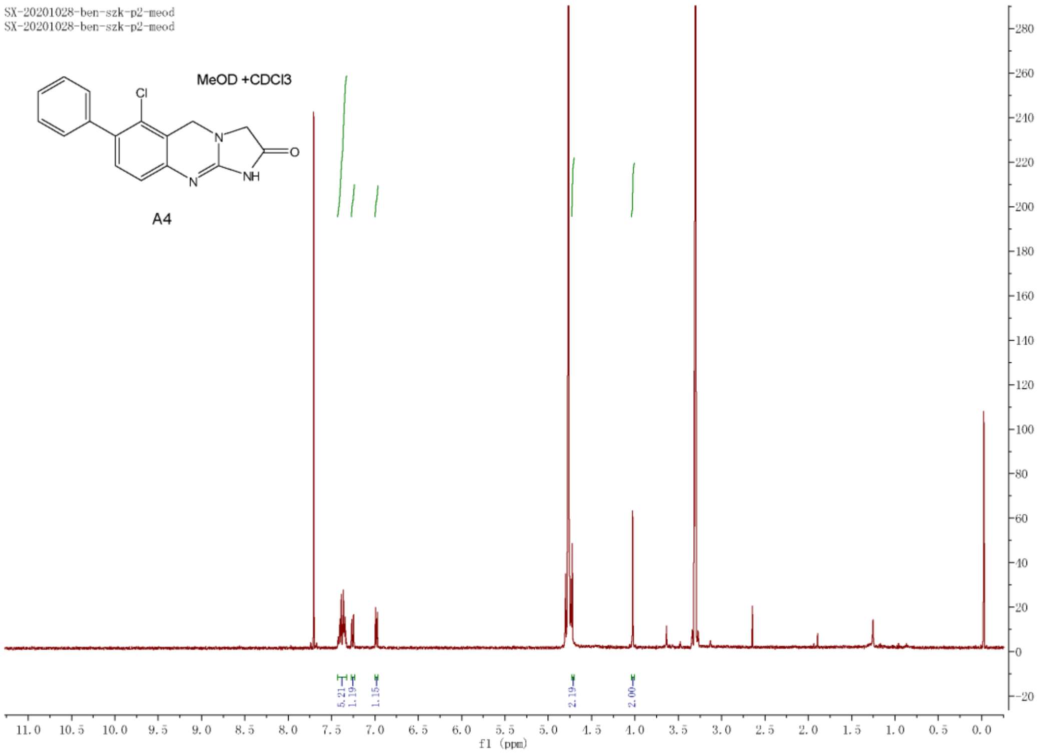
SX-20201028-ben-szk-p2-meod
SX-20201028-ben-szk-p2-meod
MeOD +CDCl3
Cl
N
NH
O
N
A4
5.21
1.19
1.15
2.19
2.00
f1 (ppm)

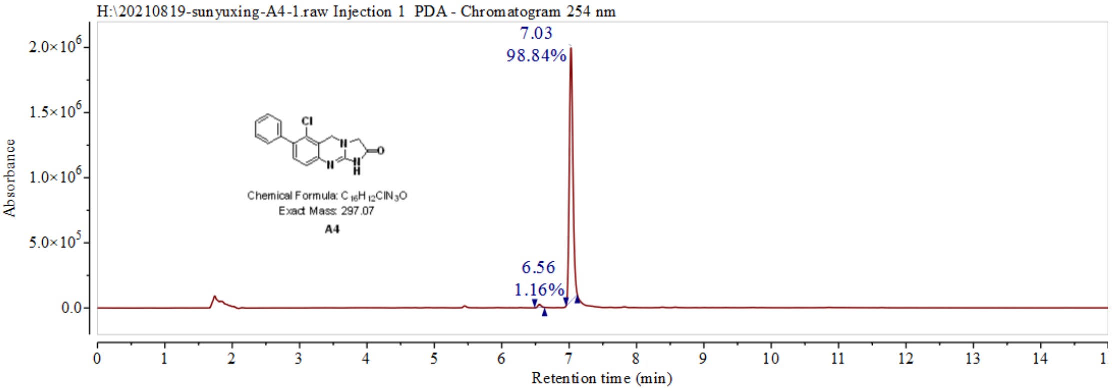
H:\20210819-sunyuxing-A4-1.raw Injection 1 PDA - Chromatogram 254 nm
7.03
98.84%
6.56
1.16%
Chemical Formula: C16H12ClN3O
Exact Mass: 297.07
A4
Absorbance
Retention time (min)

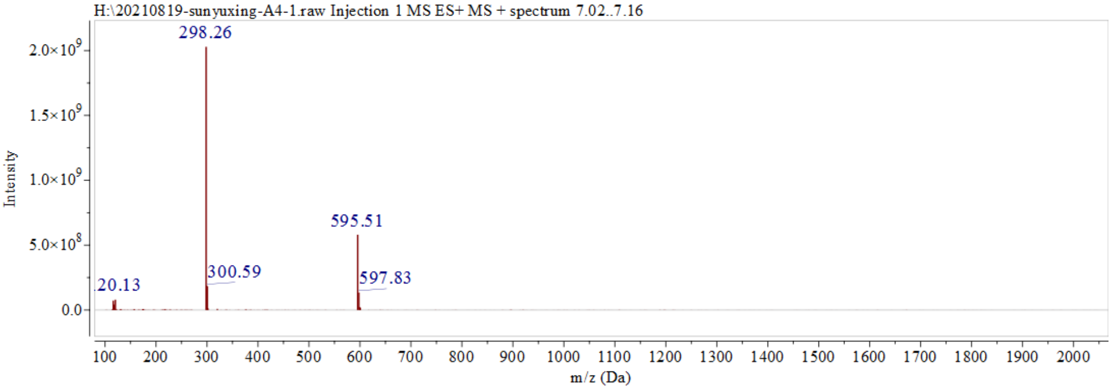
H:\20210819-sunyuxing-A4-1.raw Injection 1 MS ES+ MS + spectrum 7.02..7.16
298.26
595.51
300.59
597.83
20.13
Intensity
m/z (Da)

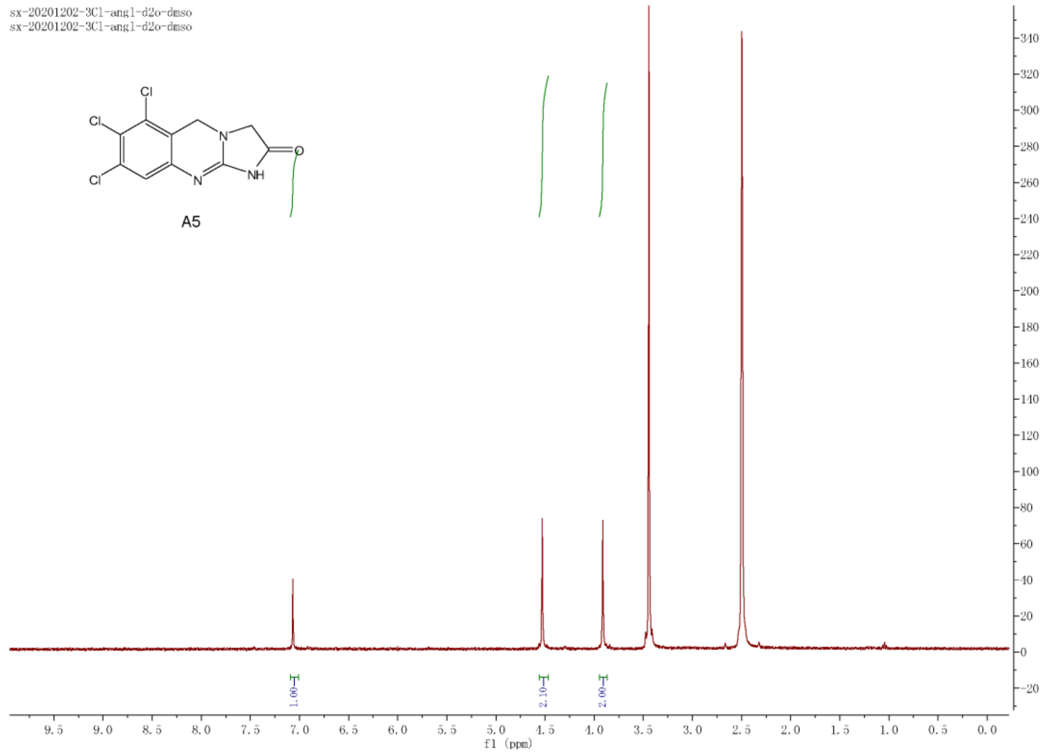
sx-20201202-3Cl-ang1-d2o-dmso
sx-20201202-3Cl-ang1-d2o-dmso
A5
1.00
2.10
2.00
f1 (ppm)

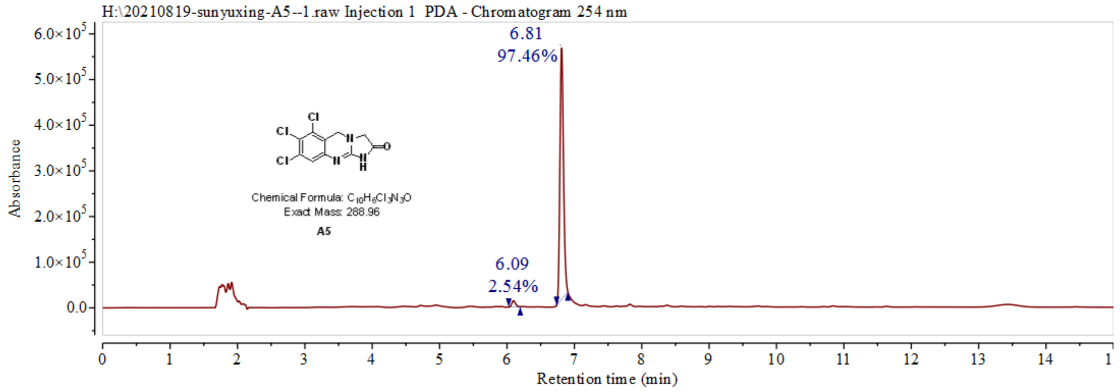
H:\20210819-sunyuxing-A5--1.raw Injection 1 PDA - Chromatogram 254 nm
6.81
97.46%
6.09
2.54%
Chemical Formula: C10H6Cl3N3O
Exact Mass: 288.96
A5
Absorbance
Retention time (min)

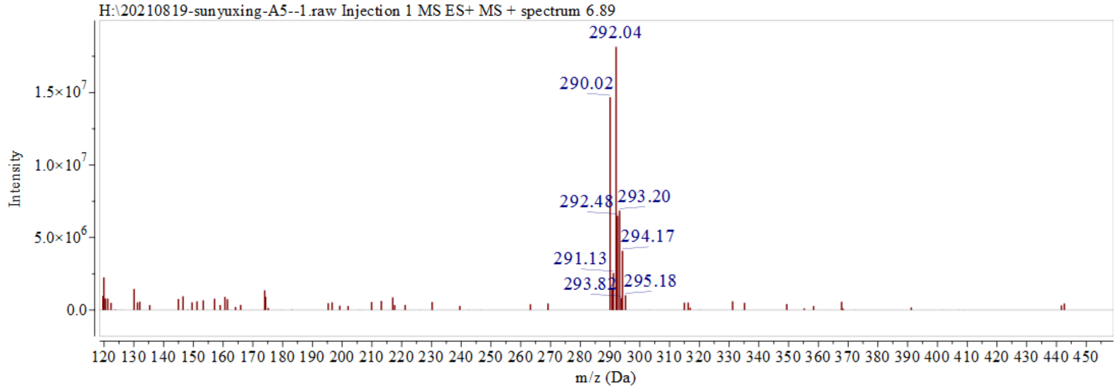
H:\20210819-sunyuxing-A5--1.raw Injection 1 MS ES+ MS + spectrum 6.89
292.04
290.02
292.48
293.20
294.17
291.13
293.82
295.18
Intensity
m/z (Da)

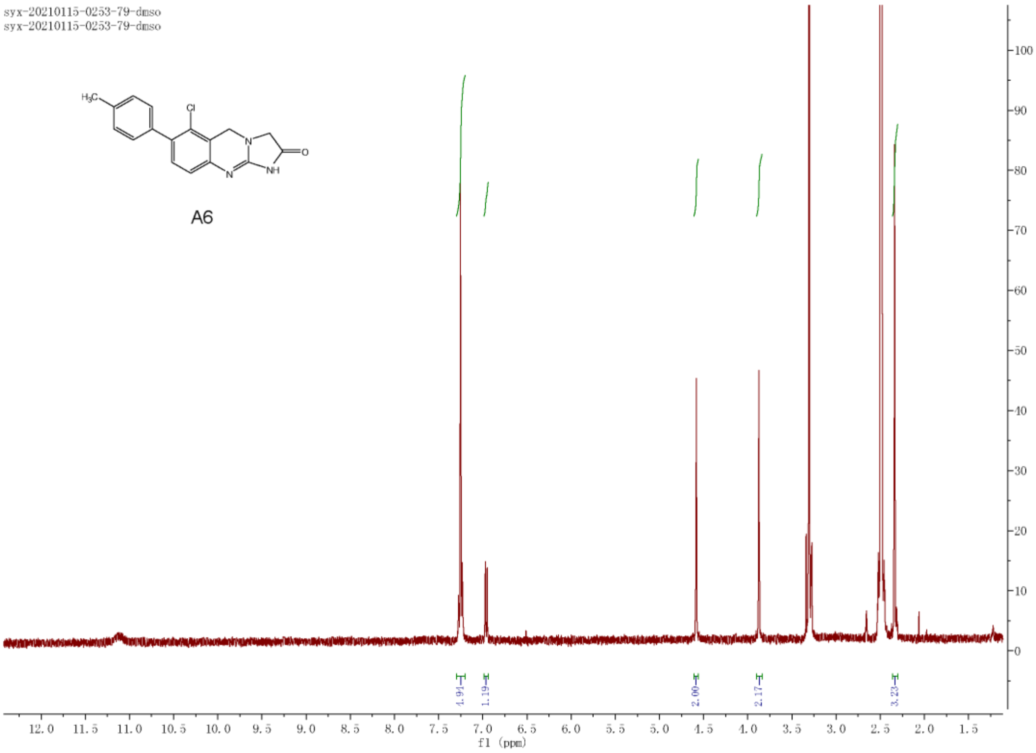
syx-20210115-0253-79-dmso
syx-20210115-0253-79-dmso
A6
4.91
1.19
2.00
2.17
3.23
f1 (ppm)

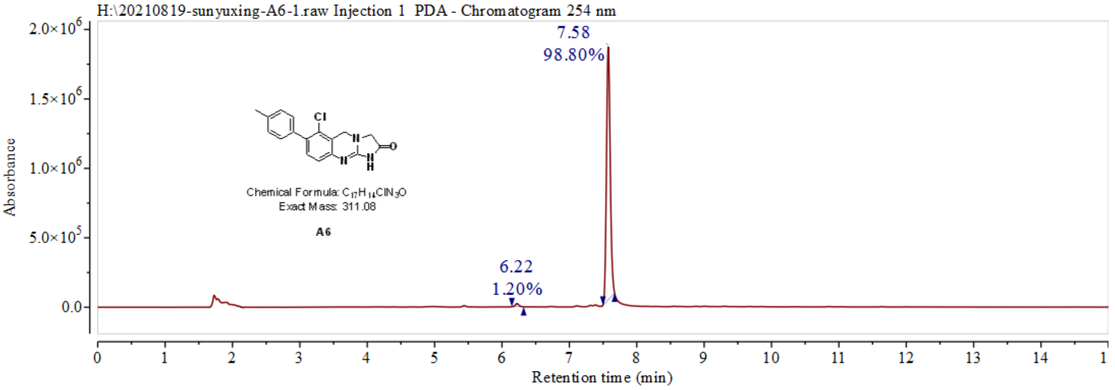
H:\20210819-sunyuxing-A6-1.raw Injection 1 PDA - Chromatogram 254 nm
7.58
98.80%
6.22
1.20%
Chemical Formula: $C_{17}H_{14}ClN_3O$
Exact Mass: 311.08
A6
Absorbance
Retention time (min)

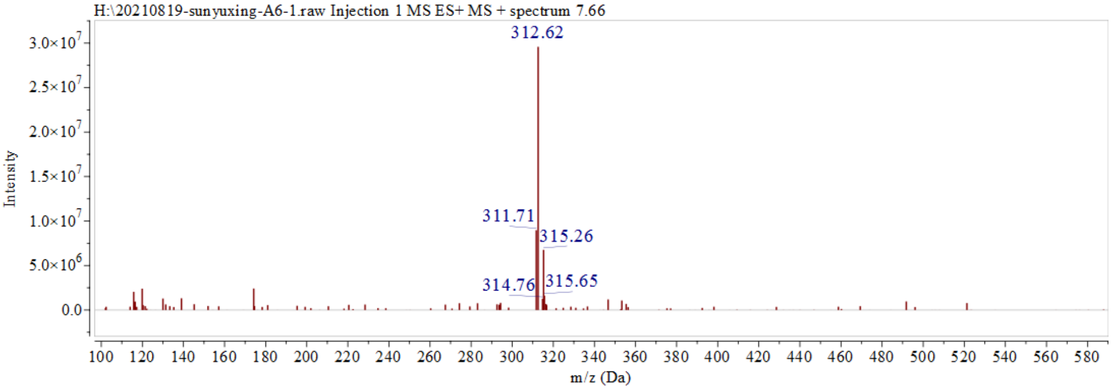
H:\20210819-sunyuxing-A6-1.raw Injection 1 MS ES+ MS + spectrum 7.66
312.62
311.71
315.26
314.76
315.65
Intensity
m/z (Da)

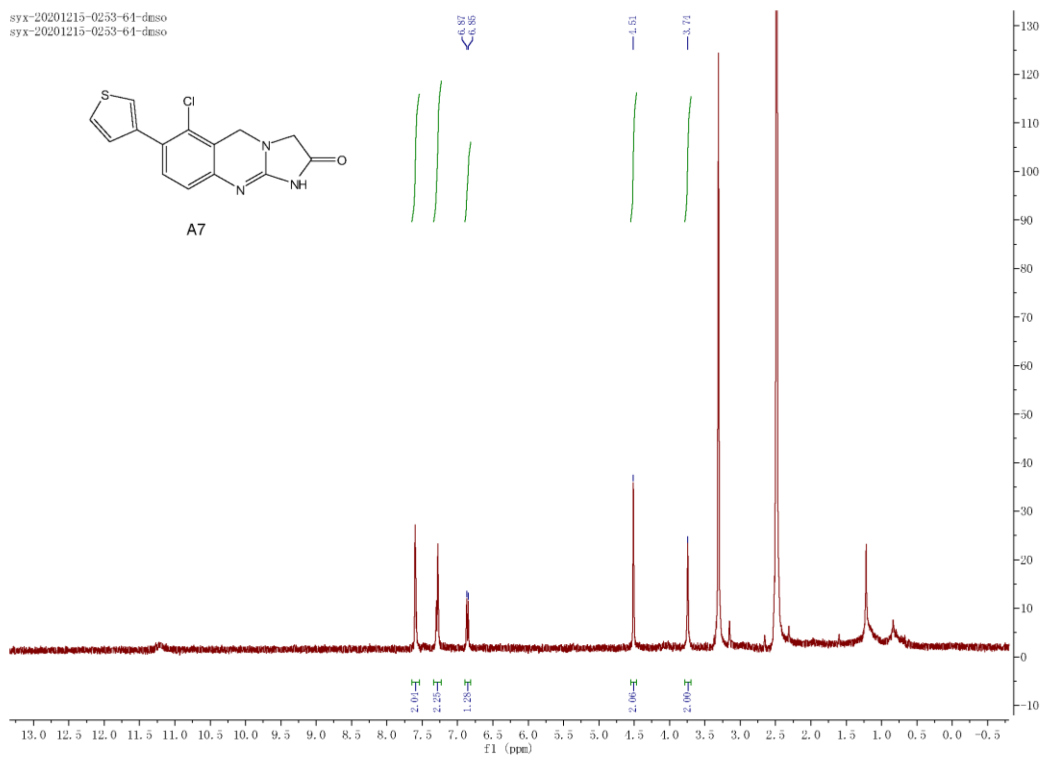

syx-20201215-0253-64-dmso
syx-20201215-0253-64-dmso
A7
6.87
6.85
4.51
3.74
2.04
2.25
1.28
2.06
2.00
f1 (ppm)

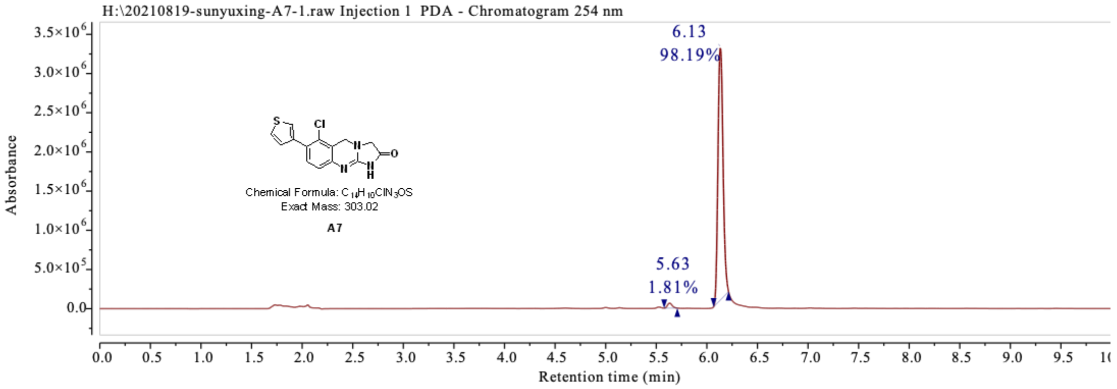

H:\20210819-sunyuxing-A7-1.raw Injection 1 PDA - Chromatogram 254 nm
6.13
98.19%
5.63
1.81%
Chemical Formula: $C_{14}H_{10}ClN_3OS$
Exact Mass: 303.02
A7
Absorbance
Retention time (min)

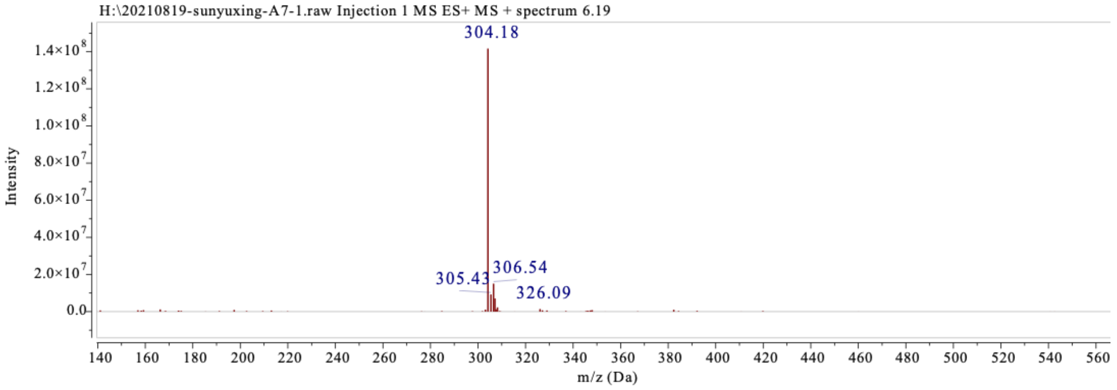

H:\20210819-sunyuxing-A7-1.raw Injection 1 MS ES+ MS + spectrum 6.19
304.18
305.43
306.54
326.09
Intensity
m/z (Da)

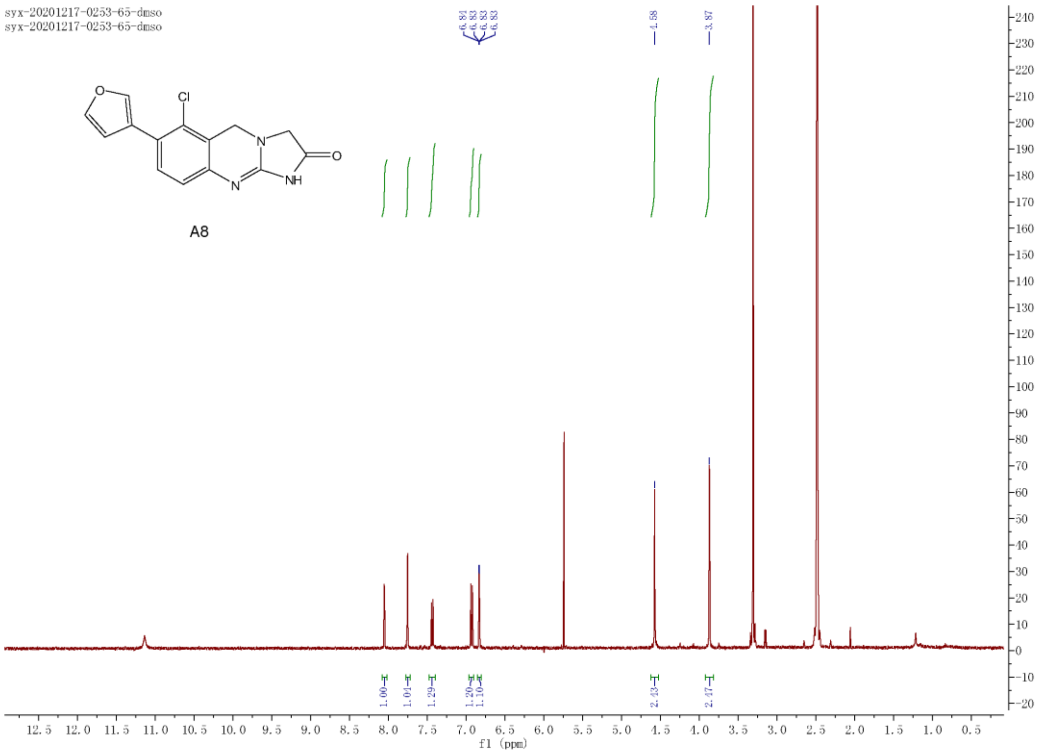

syx-20201217-0253-65-dmso
syx-20201217-0253-65-dmso
A8
f1 (ppm)

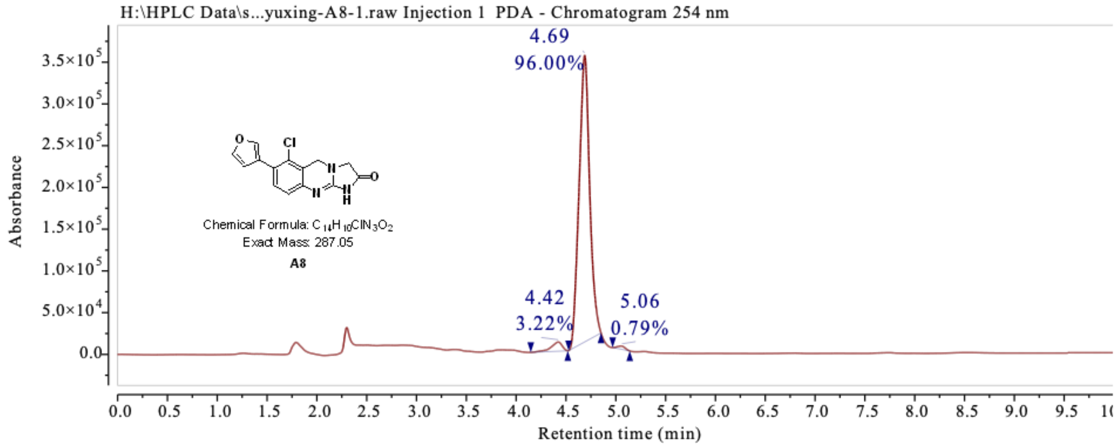

H:\HPLC Data\s...yuxing-A8-1.raw Injection 1 PDA - Chromatogram 254 nm
4.69
96.00%
4.42
3.22%
5.06
0.79%
Chemical Formula: $C_{14}H_{10}ClN_3O_2$
Exact Mass: 287.05
A8
Absorbance
Retention time (min)

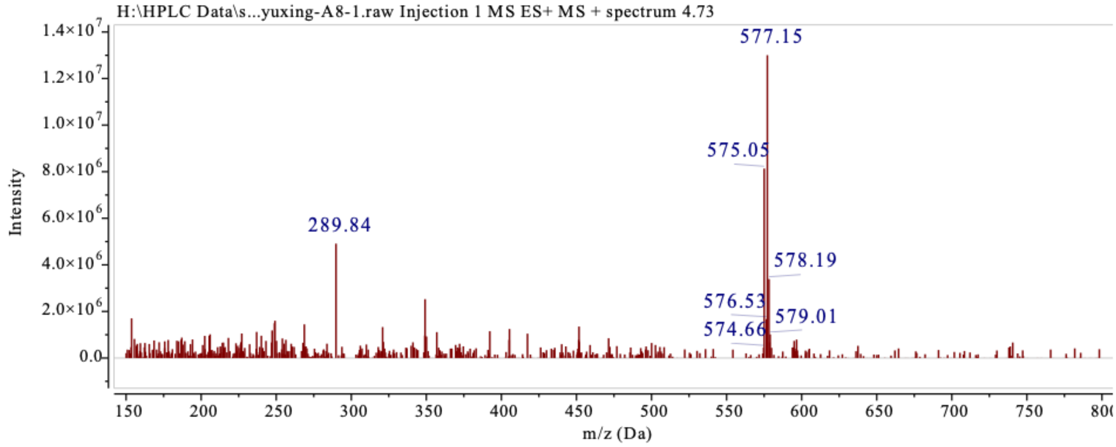

H:\HPLC Data\s...yuxing-A8-1.raw Injection 1 MS ES+ MS + spectrum 4.73
577.15
575.05
289.84
578.19
576.53
579.01
574.66
Intensity
m/z (Da)

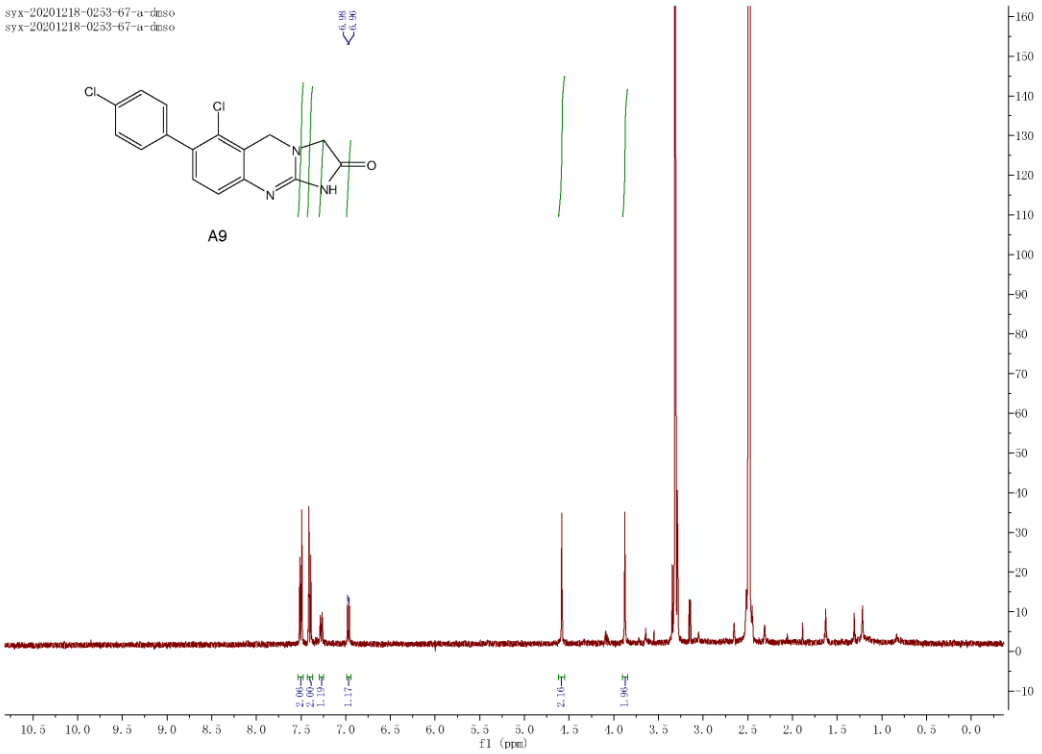
syx-20201218-0253-67-a-dmso
syx-20201218-0253-67-a-dmso
6.98
6.96
A9
2.06
2.00
1.19
1.17
2.16
1.96
f1 (ppm)

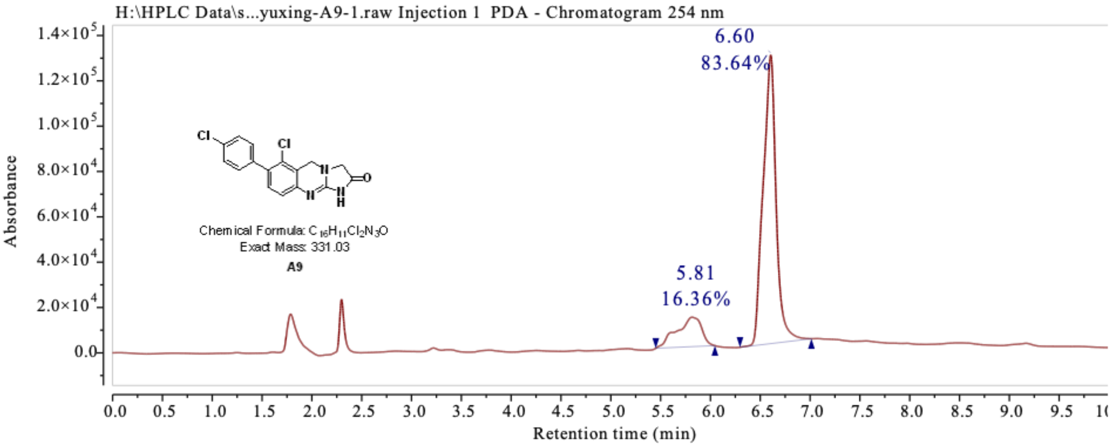
H:\HPLC Data\s...yuxing-A9-1.raw Injection 1 PDA - Chromatogram 254 nm
Chemical Formula: $C_{16}H_{11}Cl_2N_3O$
Exact Mass: 331.03
A9
6.60
83.64%
5.81
16.36%
Absorbance
Retention time (min)

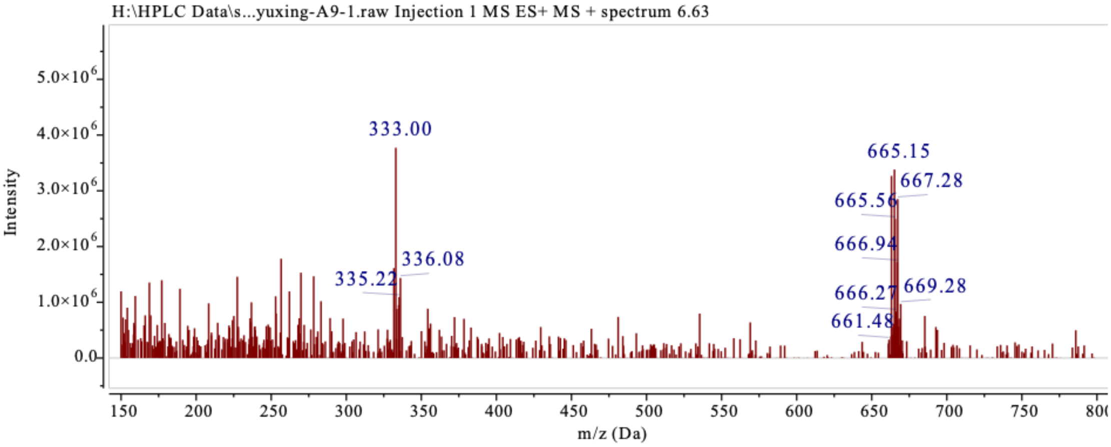
H:\HPLC Data\s...yuxing-A9-1.raw Injection 1 MS ES+ MS + spectrum 6.63
333.00
335.22
336.08
665.15
667.28
665.56
666.94
666.27
669.28
661.48
Intensity
m/z (Da)

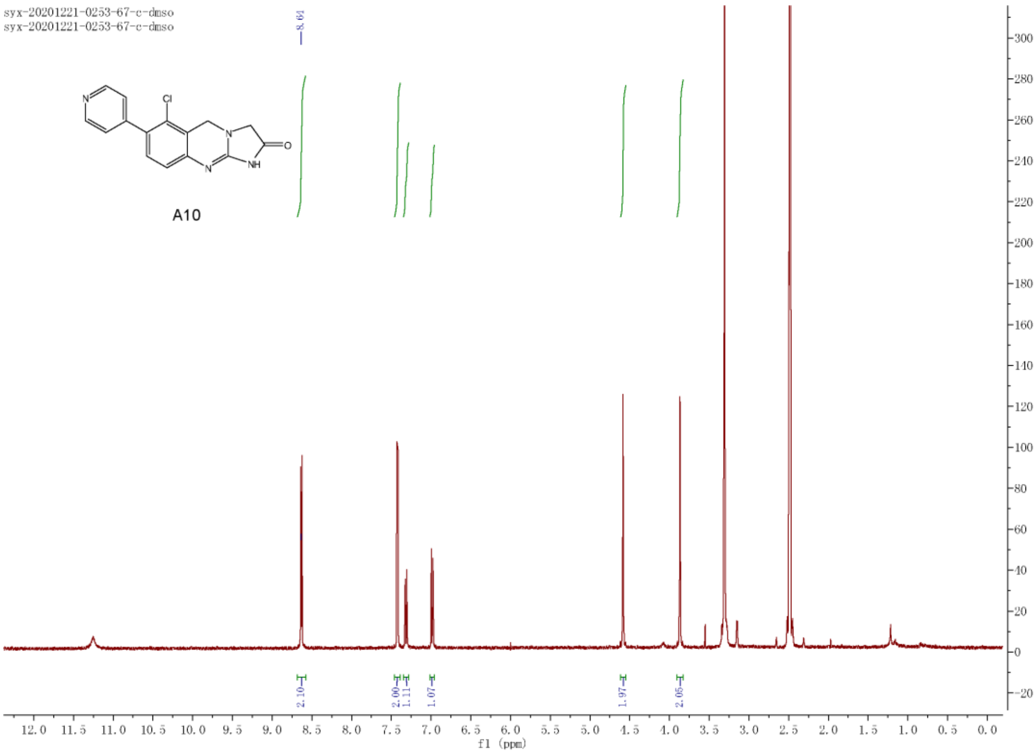
syx-20201221-0253-67-c-dmso
syx-20201221-0253-67-c-dmso
8.64
A10
2.10
2.00
1.11
1.07
1.97
2.05
f1 (ppm)

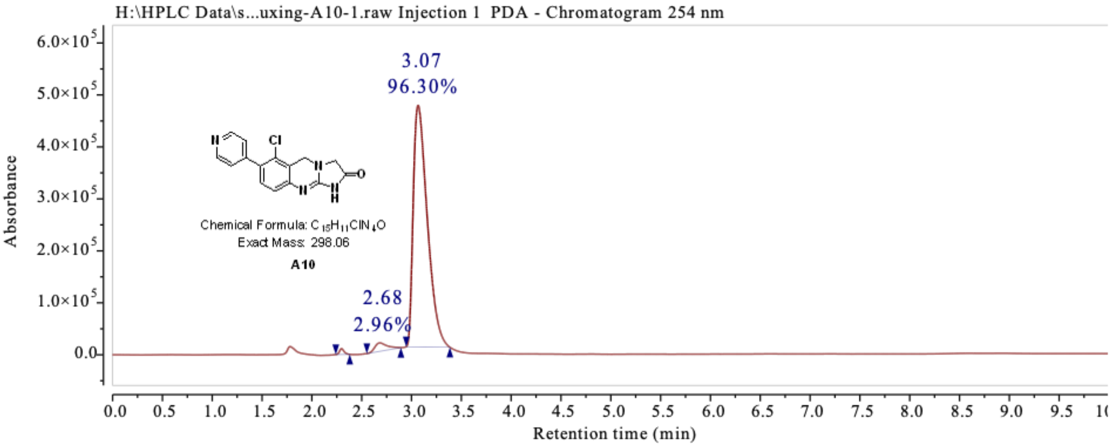
H:\HPLC Data\s...uxing-A10-1.raw Injection 1 PDA - Chromatogram 254 nm
3.07
96.30%
2.68
2.96%
Chemical Formula: C15H11ClN4O
Exact Mass: 298.06
A10
Absorbance
Retention time (min)

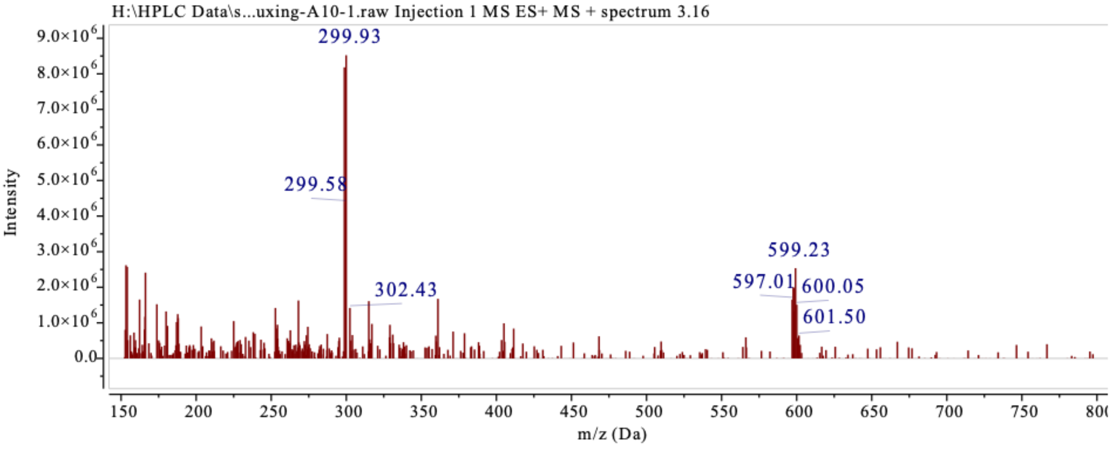
H:\HPLC Data\s...uxing-A10-1.raw Injection 1 MS ES+ MS + spectrum 3.16
299.93
299.58
302.43
599.23
597.01
600.05
601.50
Intensity
m/z (Da)

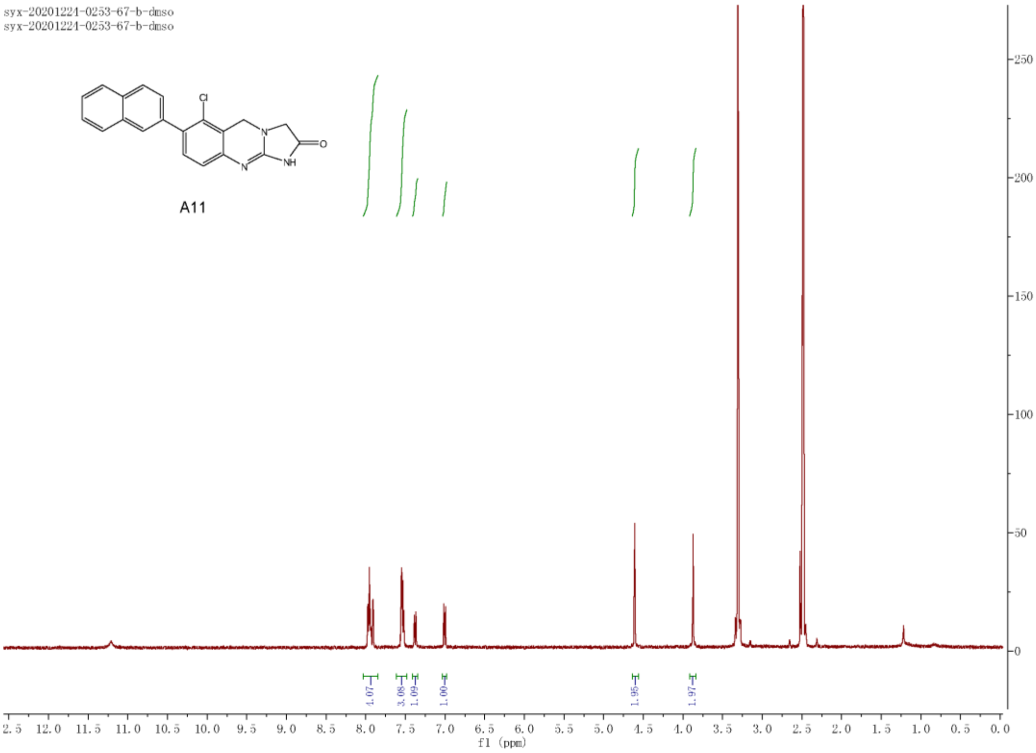

syx-20201224-0253-67-b-dmso
syx-20201224-0253-67-b-dmso
Cl
N
NH
O
A11
4.07
3.08
1.09
1.00
1.95
1.97
f1 (ppm)

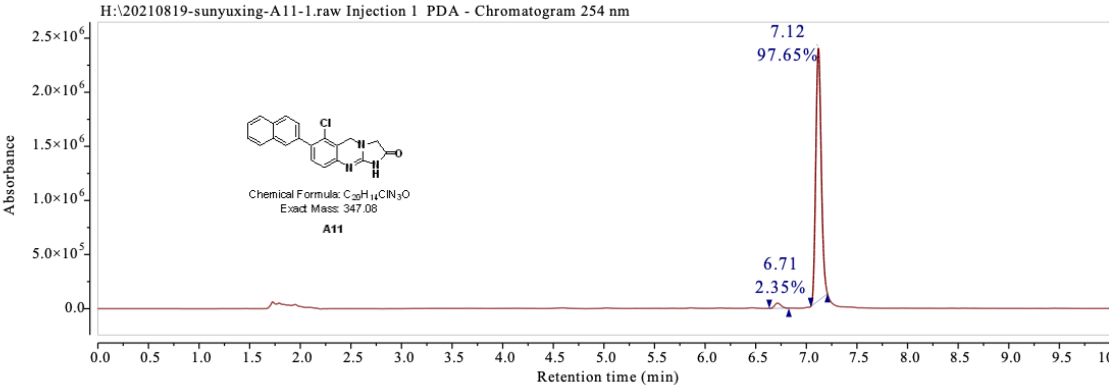

H:\20210819-sunyuxing-A11-1.raw Injection 1 PDA - Chromatogram 254 nm
7.12
97.65%
6.71
2.35%
Chemical Formula: C20H14ClN3O
Exact Mass: 347.08
A11
Absorbance
Retention time (min)

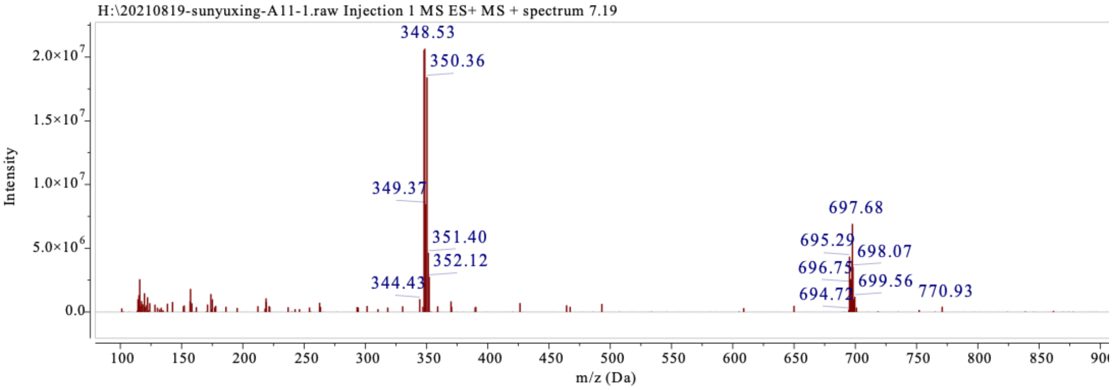

H:\20210819-sunyuxing-A11-1.raw Injection 1 MS ES+ MS + spectrum 7.19
348.53
350.36
349.37
351.40
352.12
344.43
697.68
695.29
698.07
696.75
699.56
694.72
770.93
Intensity
m/z (Da)

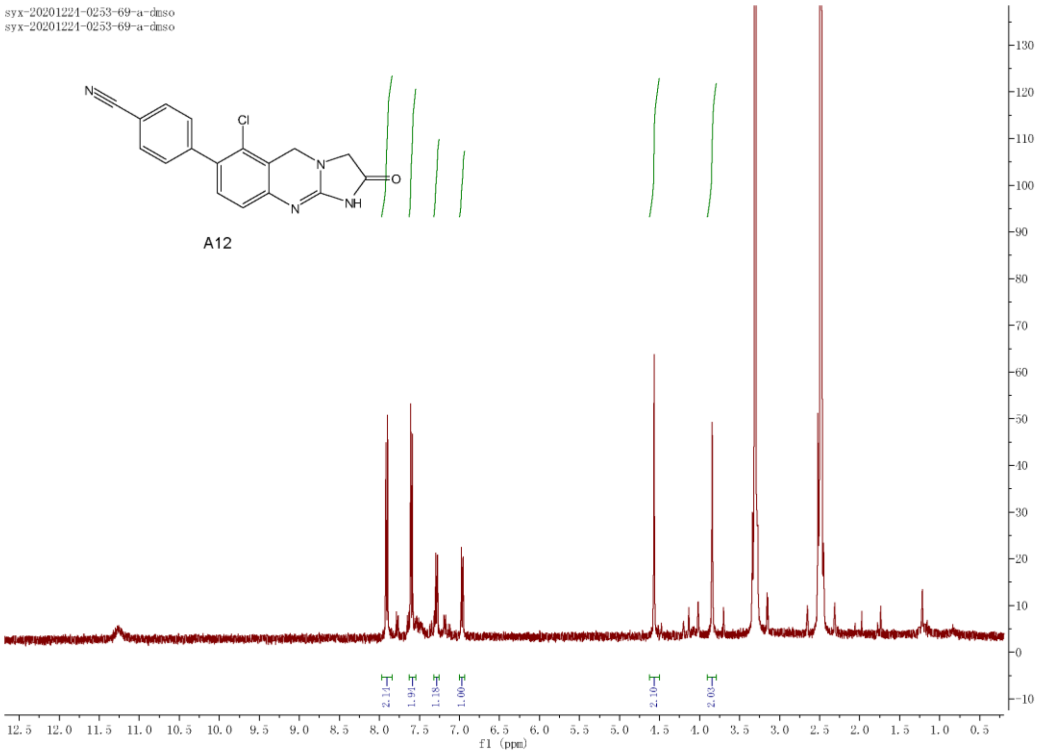
syx-20201224-0253-69-a-dmso
syx-20201224-0253-69-a-dmso
A12
2.14
1.94
1.18
1.00
2.10
2.03
f1 (ppm)

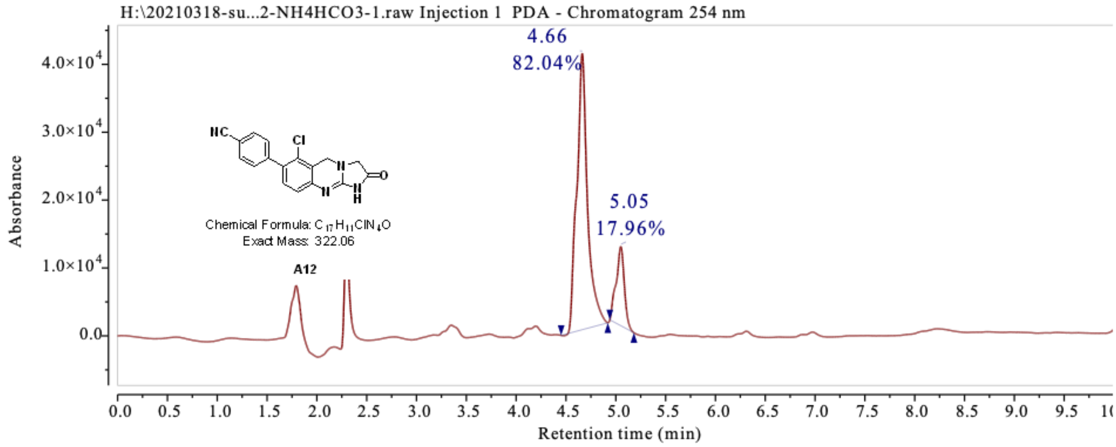
H:\20210318-su...2-NH4HCO3-1.raw Injection 1 PDA - Chromatogram 254 nm
4.66
82.04%
5.05
17.96%
Chemical Formula: C17H11ClN4O
Exact Mass: 322.06
A12
Absorbance
Retention time (min)

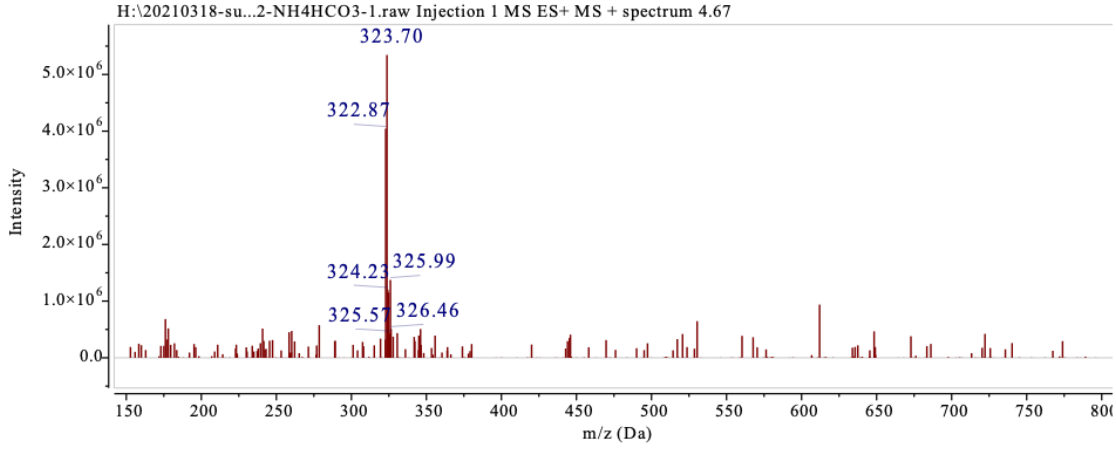
H:\20210318-su...2-NH4HCO3-1.raw Injection 1 MS ES+ MS + spectrum 4.67
323.70
322.87
324.23
325.99
325.57
326.46
Intensity
m/z (Da)

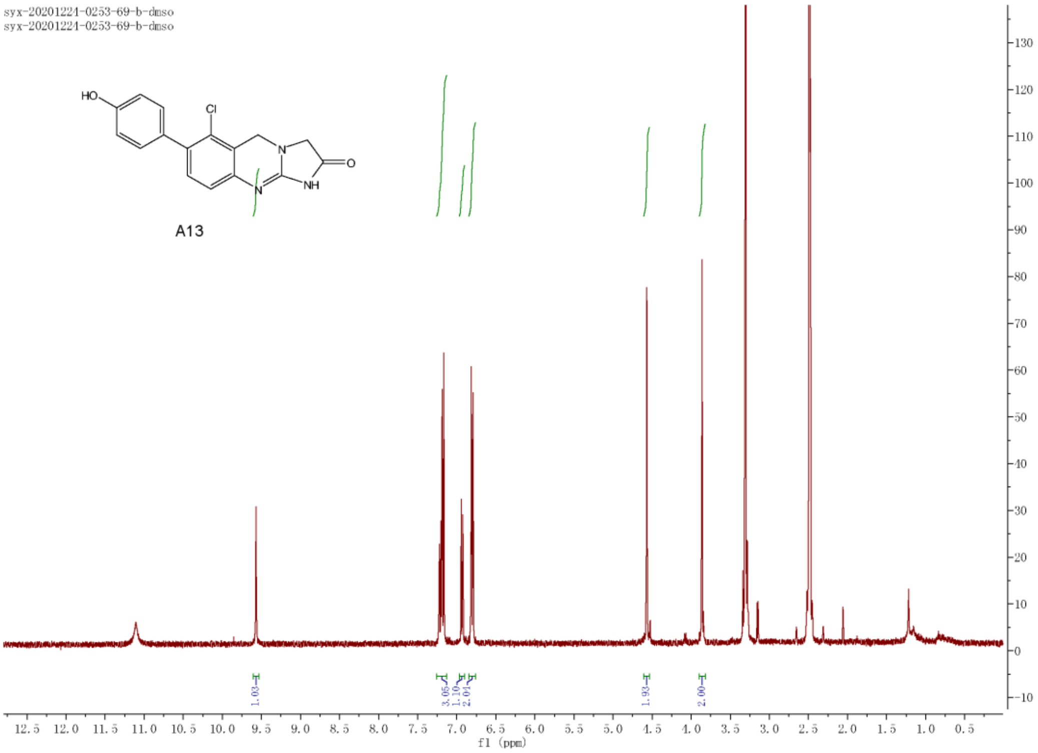
syx-20201224-0253-69-b-dmso
syx-20201224-0253-69-b-dmso
HO
Cl
N
N
NH
O
A13
1.03
3.05
1.10
2.04
1.93
2.00
f1 (ppm)

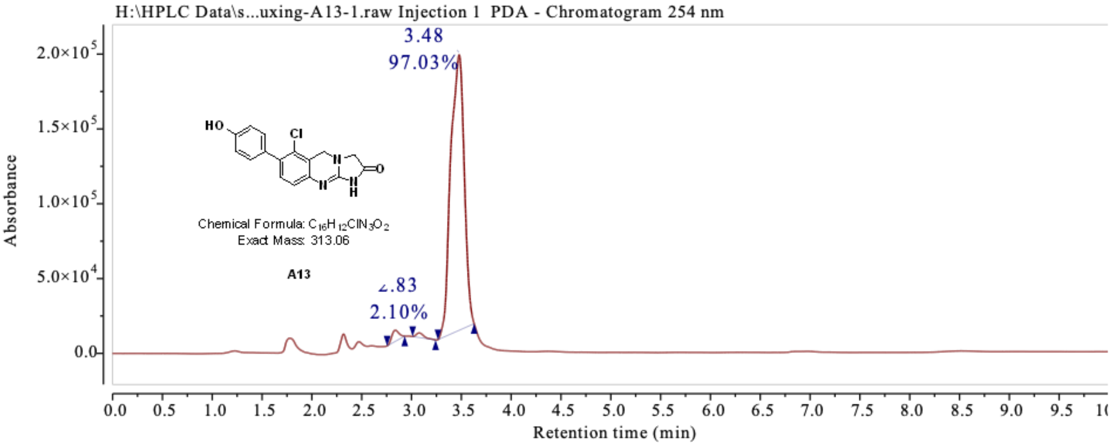
H:\HPLC Data\s...uxing-A13-1.raw Injection 1 PDA - Chromatogram 254 nm
3.48
97.03%
2.10%
HO
Cl
N
N
H
O
Chemical Formula: C16H12ClN3O2
Exact Mass: 313.06
A13
Absorbance
Retention time (min)

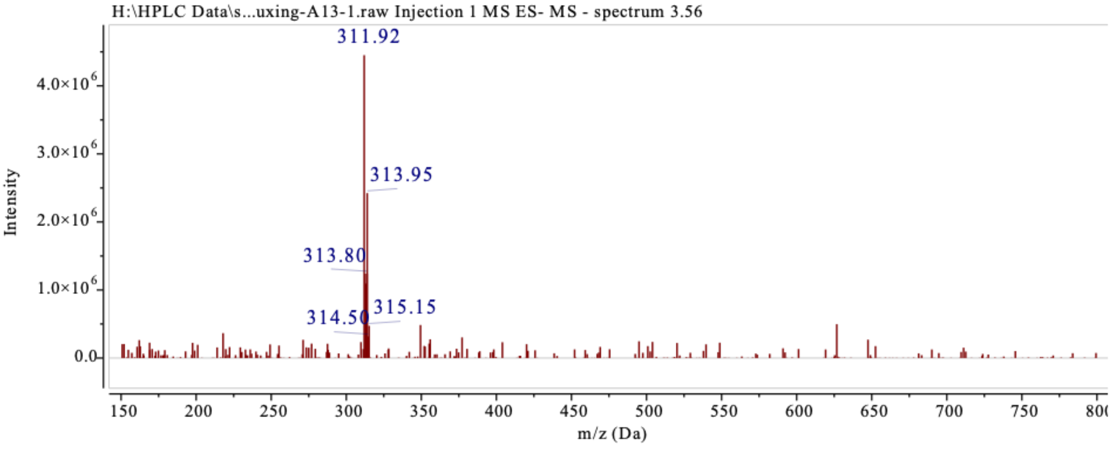
H:\HPLC Data\s...uxing-A13-1.raw Injection 1 MS ES- MS - spectrum 3.56
311.92
313.95
313.80
314.50
315.15
Intensity
m/z (Da)

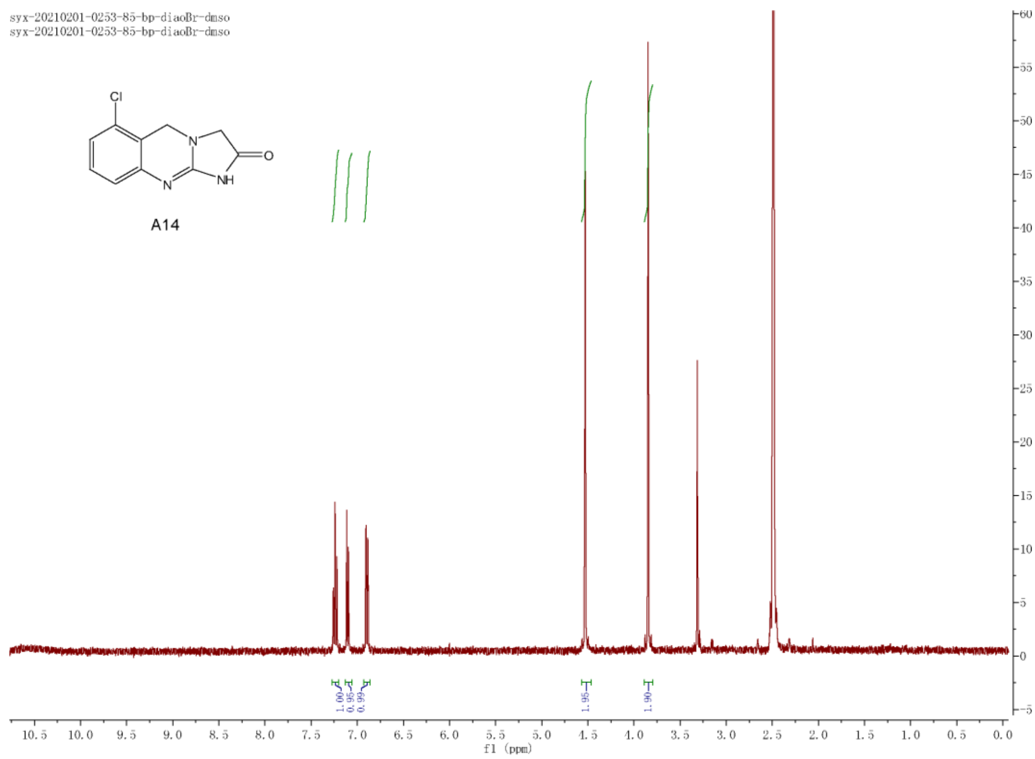
syx-20210201-0253-85-bp-diaoBr-dmso
syx-20210201-0253-85-bp-diaoBr-dmso
Cl
N
N
NH
O
A14
1.00
0.95
0.99
1.95
1.90
10.5 10.0 9.5 9.0 8.5 8.0 7.5 7.0 6.5 6.0 5.5 5.0 4.5 4.0 3.5 3.0 2.5 2.0 1.5 1.0 0.5 0.0
f1 (ppm)
60 55 50 45 40 35 30 25 20 15 10 5 0 -5

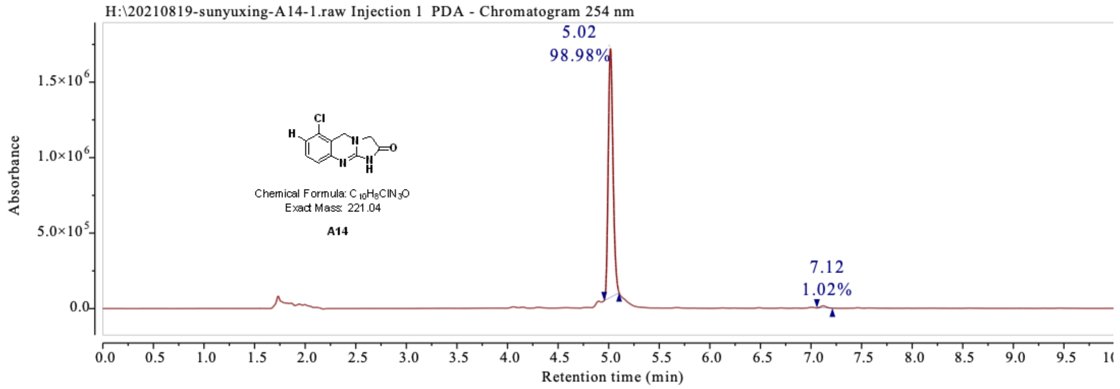
H:\20210819-sunyuxing-A14-1.raw Injection 1 PDA - Chromatogram 254 nm
5.02
98.98%
7.12
1.02%
Chemical Formula: $C_{10}H_8ClN_3O$
Exact Mass: 221.04
A14
Absorbance
Retention time (min)

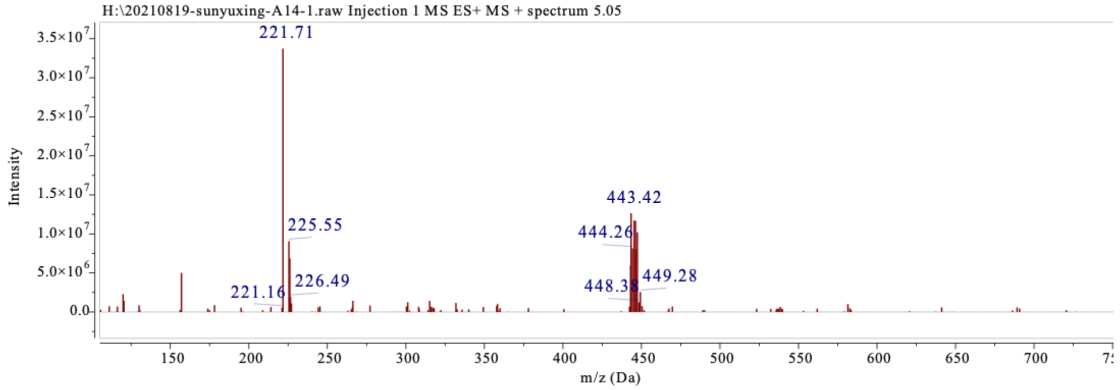
H:\20210819-sunyuxing-A14-1.raw Injection 1 MS ES+ MS + spectrum 5.05
221.71
225.55
226.49
221.16
443.42
444.26
448.38
449.28
Intensity
m/z (Da)

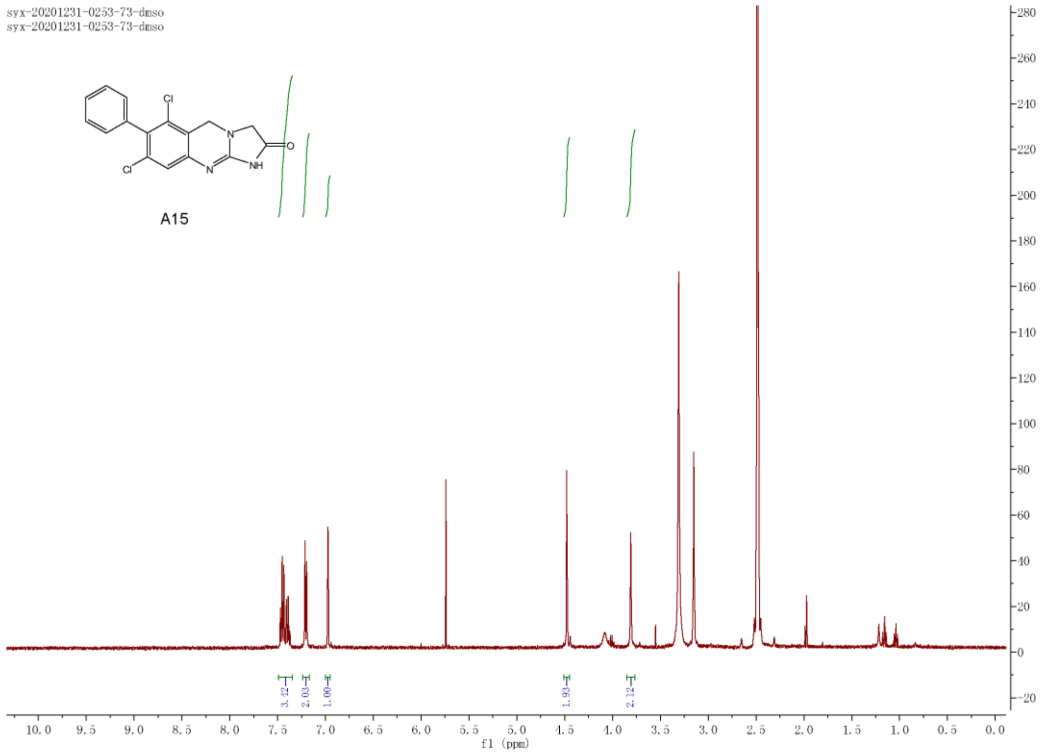
syx-20201231-0253-73-dmso
syx-20201231-0253-73-dmso
Cl
Cl
N
N
NH
O
A15
3.42
2.03
1.00
1.93
2.12
f1 (ppm)

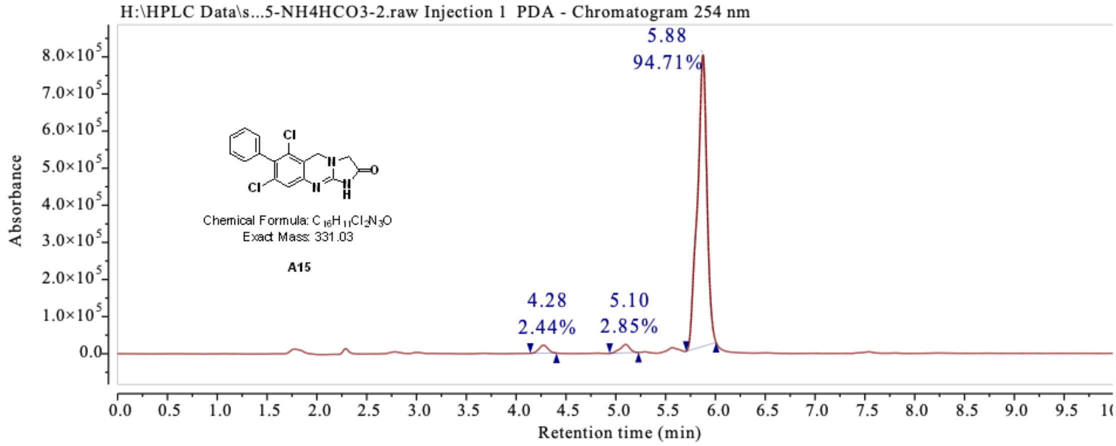
H:\HPLC Data\s...5-NH4HCO3-2.raw Injection 1 PDA - Chromatogram 254 nm
Absorbance
Retention time (min)
5.88
94.71%
4.28
2.44%
5.10
2.85%
Chemical Formula: C16H11Cl2N3O
Exact Mass: 331.03
A15

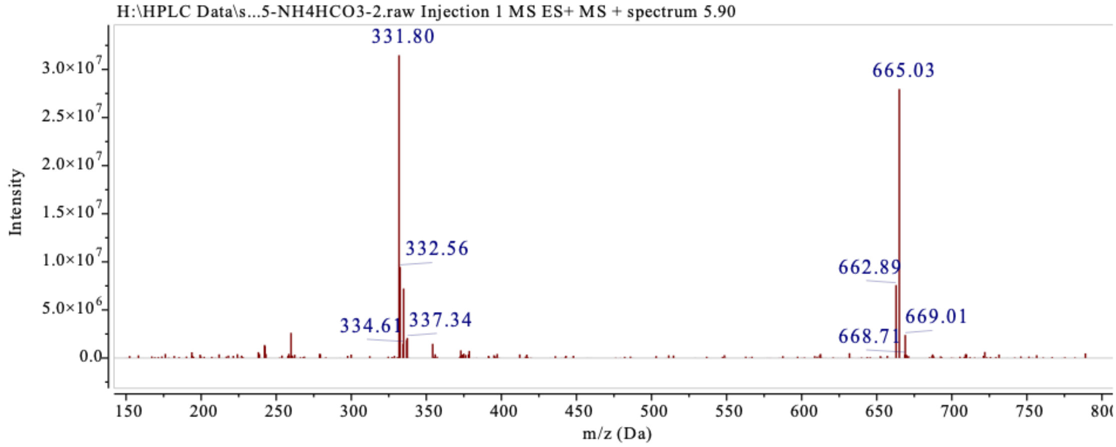
H:\HPLC Data\s...5-NH4HCO3-2.raw Injection 1 MS ES+ MS + spectrum 5.90
Intensity
m/z (Da)
331.80
332.56
334.61
337.34
665.03
662.89
669.01
668.71

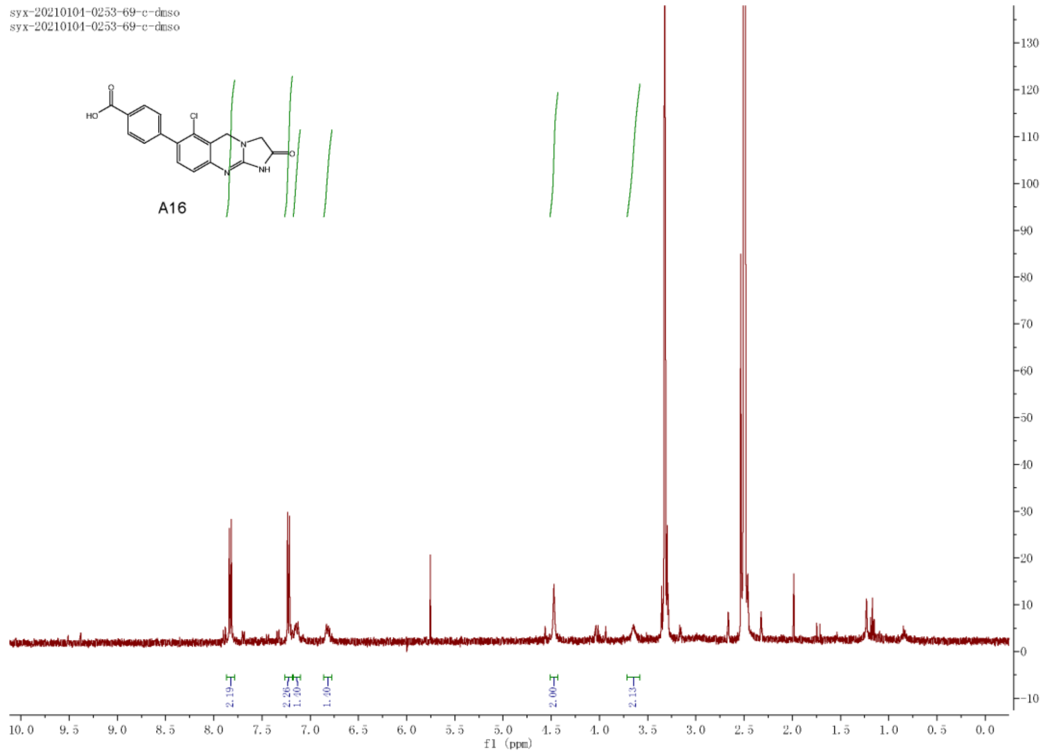
syx-20210101-0253-69-c-dmso
syx-20210101-0253-69-c-dmso
HO
O
Cl
N
NH
O
A16
2.19
2.26
1.10
1.10
2.00
2.13
f1 (ppm)

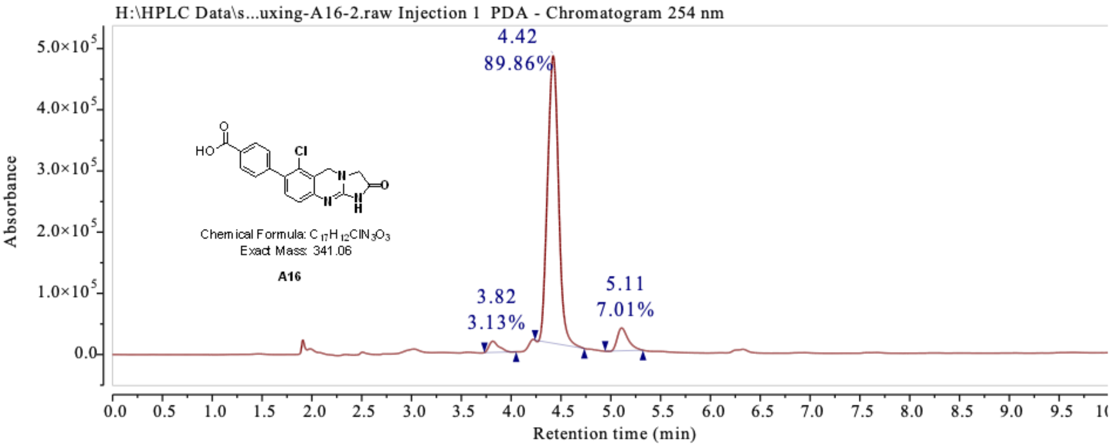
H:\HPLC Data\s...uxing-A16-2.raw Injection 1 PDA - Chromatogram 254 nm
4.42
89.86%
3.82
3.13%
5.11
7.01%
Chemical Formula: $C_{17}H_{12}ClN_3O_3$
Exact Mass: 341.06
A16
Absorbance
Retention time (min)

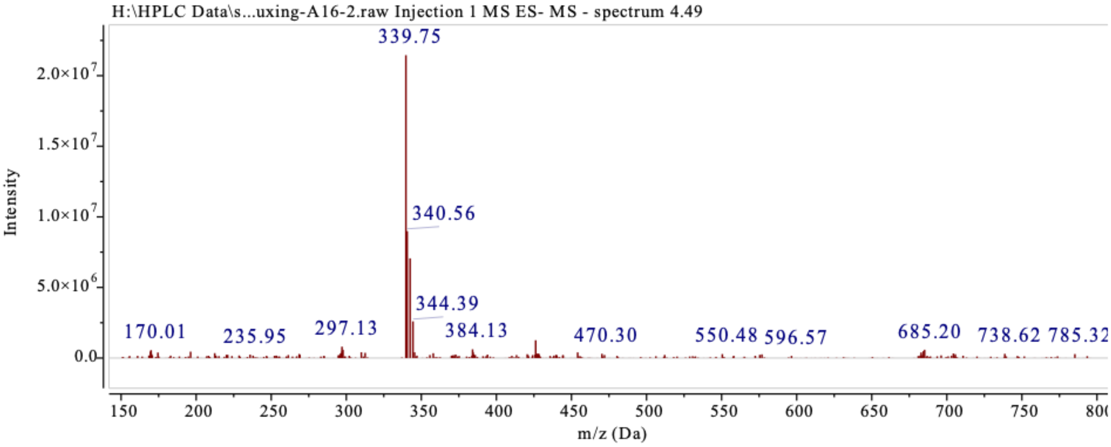
H:\HPLC Data\s...uxing-A16-2.raw Injection 1 MS ES- MS - spectrum 4.49
339.75
340.56
344.39
384.13
170.01
235.95
297.13
470.30
550.48
596.57
685.20
738.62
785.32
Intensity
m/z (Da)

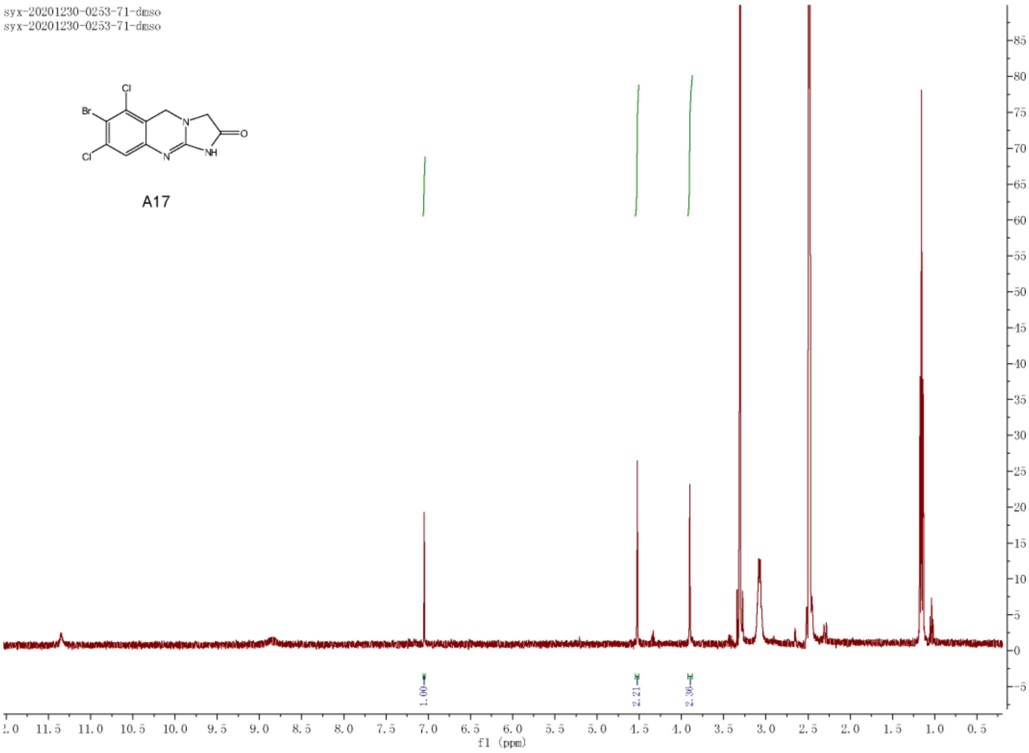
syx-20201230-0253-71-dmso
syx-20201230-0253-71-dmso
Br
Cl
Cl
N
N
NH
O
A17
1.00
2.21
2.36
f1 (ppm)

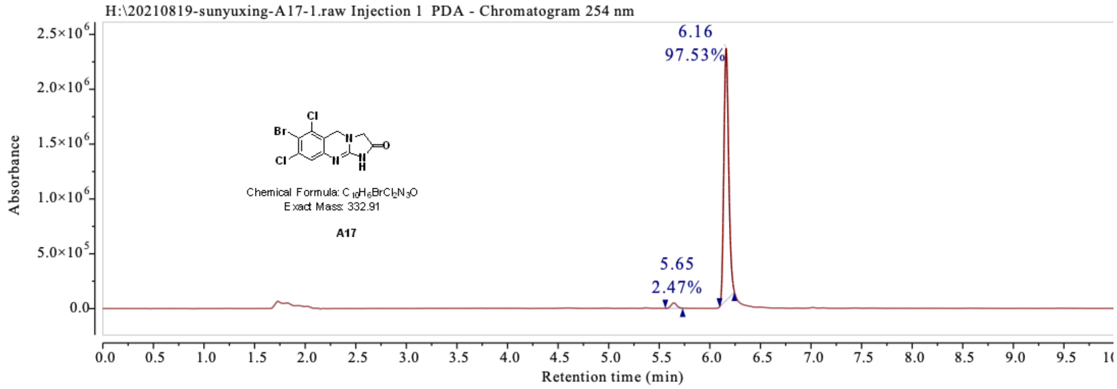
H:\20210819-sunyuxing-A17-1.raw Injection 1 PDA - Chromatogram 254 nm
6.16
97.53%
5.65
2.47%
Absorbance
Retention time (min)
Chemical Formula: $C_{10}H_6BrCl_2N_3O$
Exact Mass: 332.91
A17

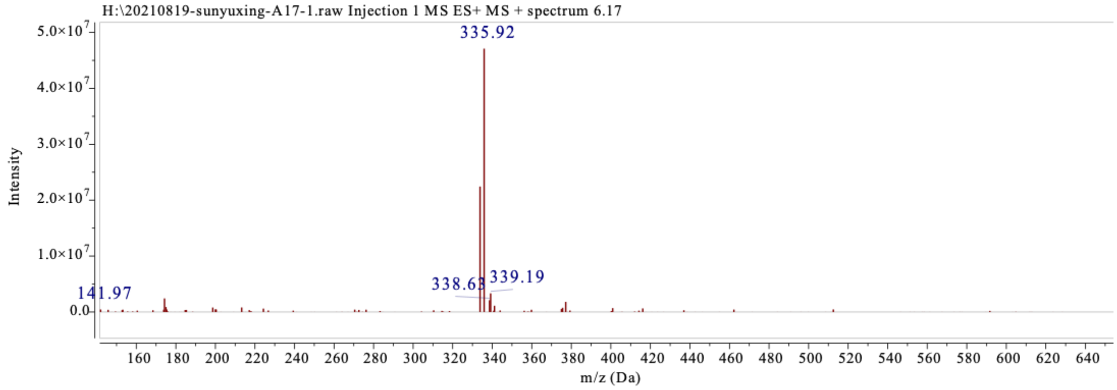
H:\20210819-sunyuxing-A17-1.raw Injection 1 MS ES+ MS + spectrum 6.17
335.92
338.63
339.19
141.97
Intensity
m/z (Da)

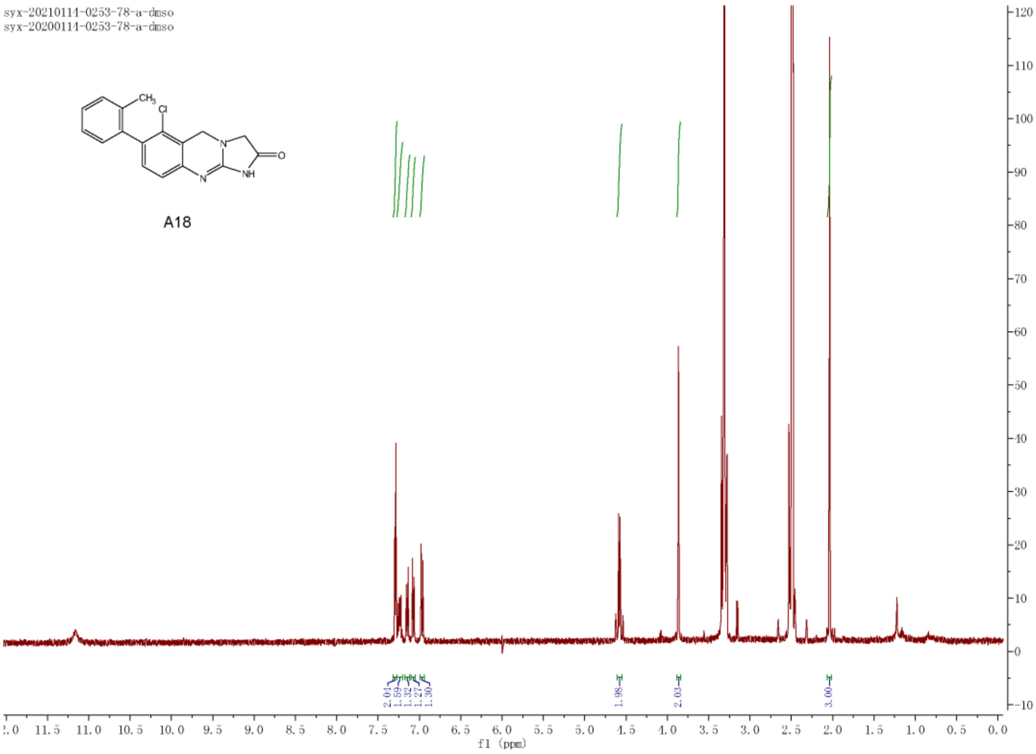
syx-20210114-0253-78-a-dmso
syx-20200114-0253-78-a-dmso
A18
2.04
1.59
1.32
1.27
1.30
1.98
2.03
3.00
f1 (ppm)

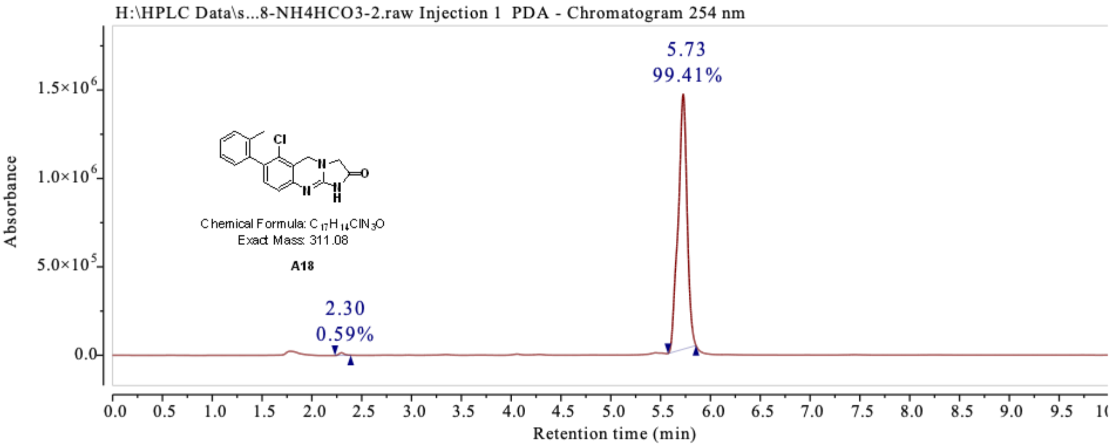
H:\HPLC Data\s...8-NH4HCO3-2.raw Injection 1 PDA - Chromatogram 254 nm
5.73
99.41%
2.30
0.59%
Chemical Formula: $C_{17}H_{14}ClN_3O$
Exact Mass: 311.08
A18
Absorbance
Retention time (min)

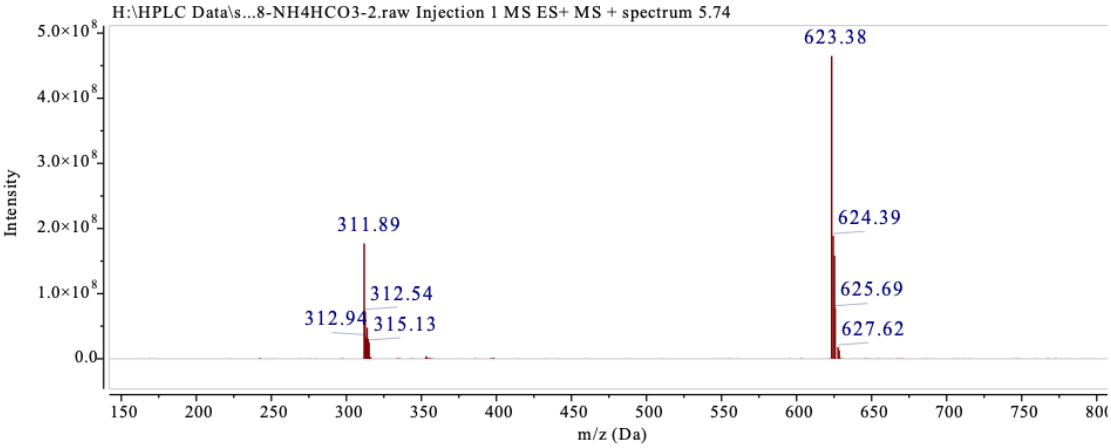
H:\HPLC Data\s...8-NH4HCO3-2.raw Injection 1 MS ES+ MS + spectrum 5.74
623.38
624.39
625.69
627.62
311.89
312.54
312.94
315.13
Intensity
m/z (Da)

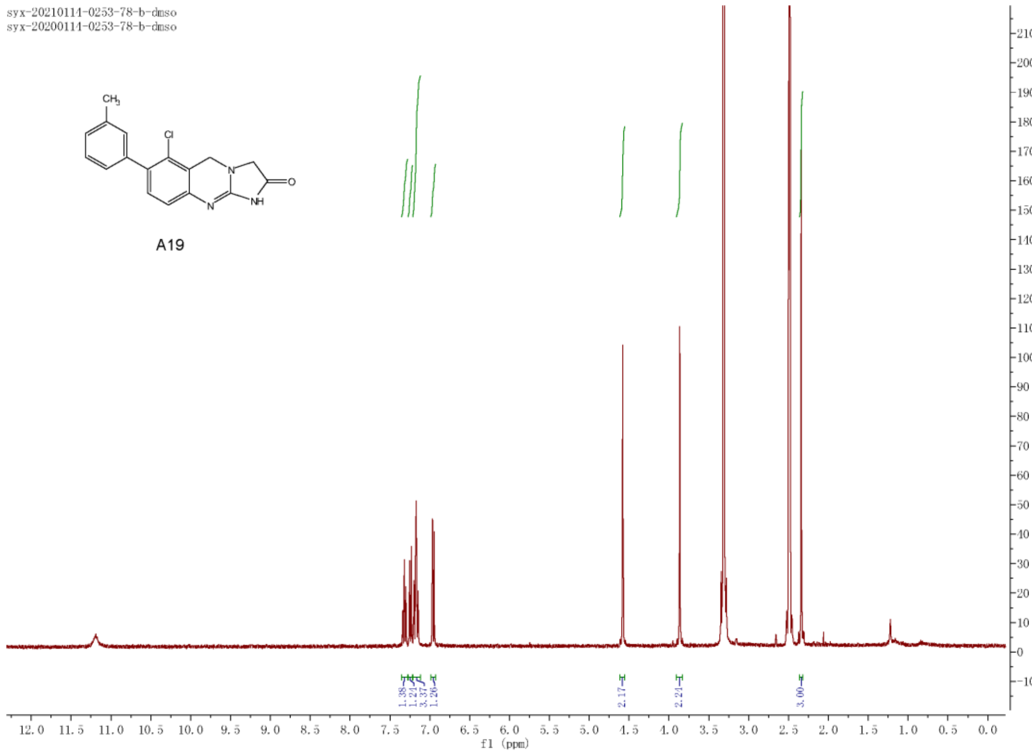
syx-20210114-0253-78-b-dmso
syx-20200114-0253-78-b-dmso
A19
f1 (ppm)

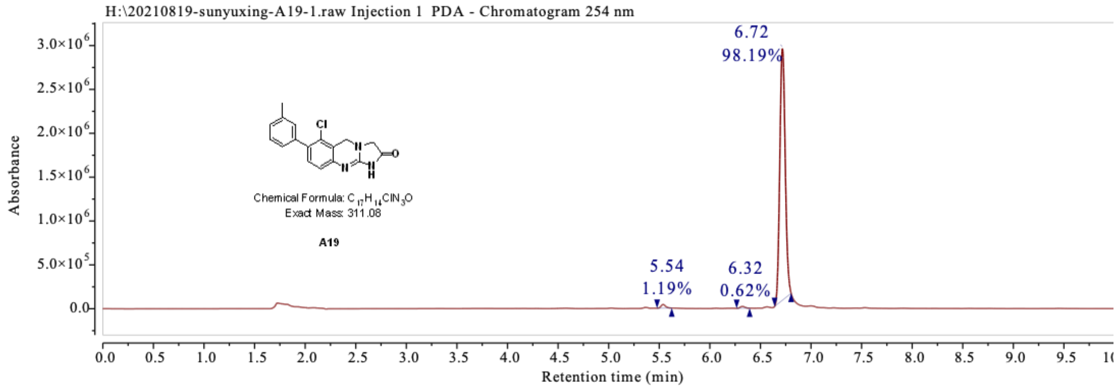
H:\20210819-sunyuxing-A19-1.raw Injection 1 PDA - Chromatogram 254 nm
Chemical Formula: C17H14ClN3O
Exact Mass: 311.08
A19
6.72
98.19%
5.54
1.19%
6.32
0.62%
Absorbance
Retention time (min)

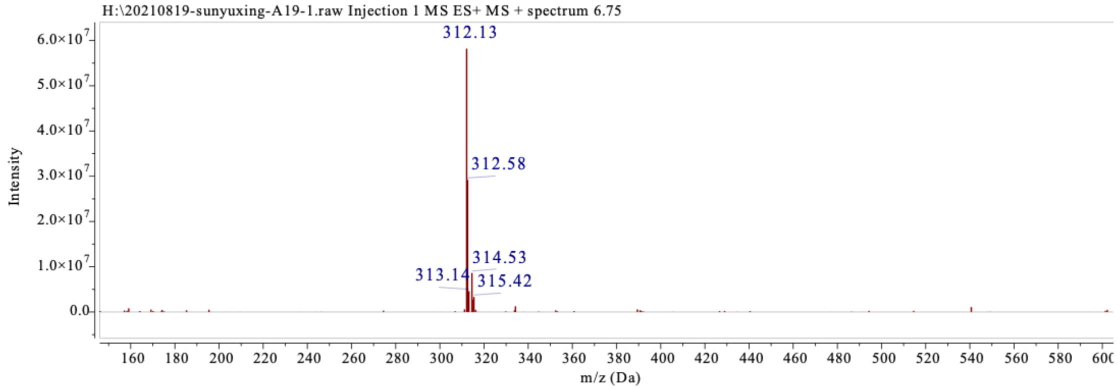
H:\20210819-sunyuxing-A19-1.raw Injection 1 MS ES+ MS + spectrum 6.75
312.13
312.58
314.53
313.14
315.42
Intensity
m/z (Da)

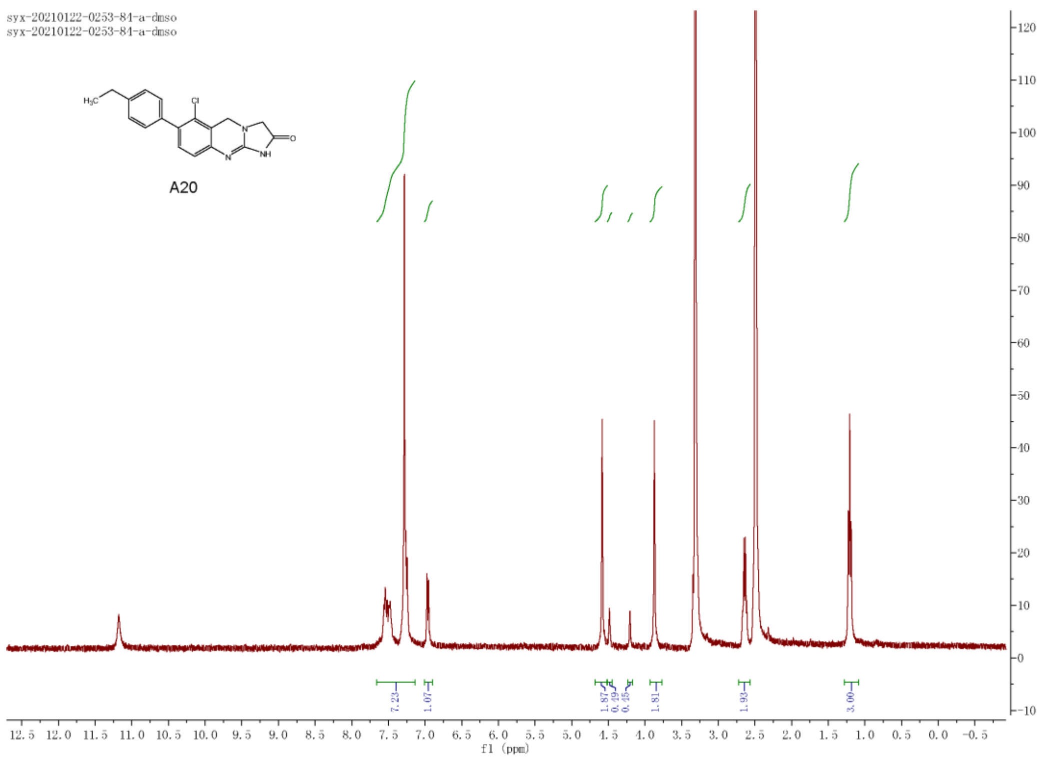
syx-20210122-0253-84-a-dmso
syx-20210122-0253-84-a-dmso
A20
7.23
1.07
1.87
0.19
0.25
1.81
1.93
3.00
f1 (ppm)

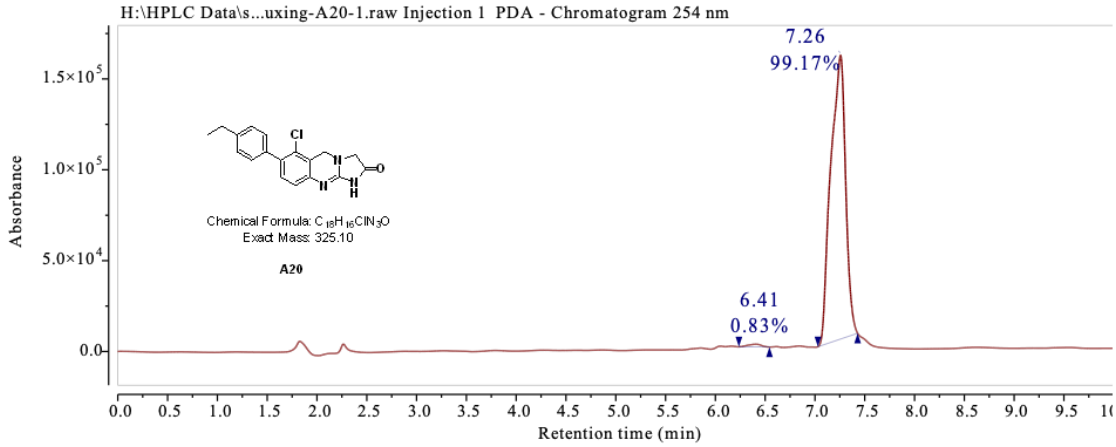
H:\HPLC Data\s...uxing-A20-1.raw Injection 1 PDA - Chromatogram 254 nm
7.26
99.17%
6.41
0.83%
Chemical Formula: C18H16ClN3O
Exact Mass: 325.10
A20
Absorbance
Retention time (min)

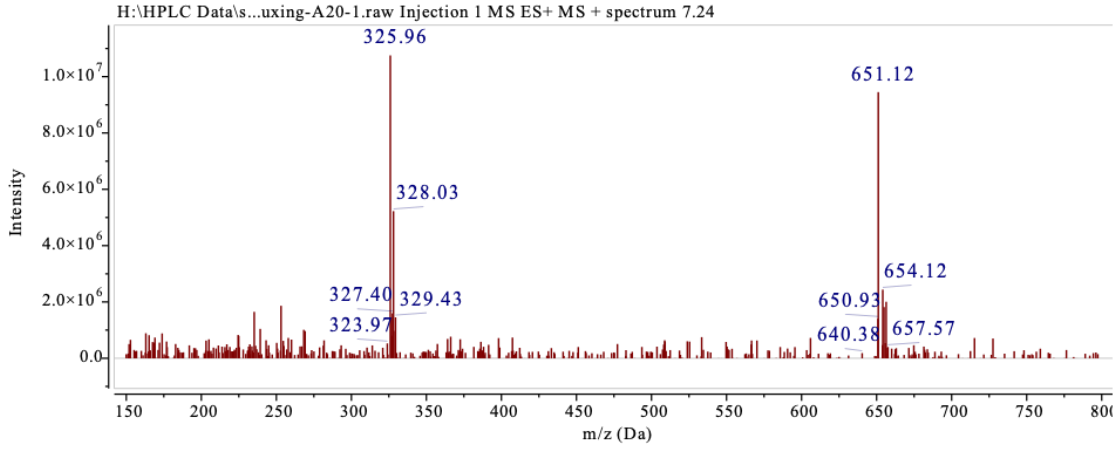
H:\HPLC Data\s...uxing-A20-1.raw Injection 1 MS ES+ MS + spectrum 7.24
325.96
328.03
327.40
329.43
323.97
651.12
654.12
650.93
640.38
657.57
Intensity
m/z (Da)

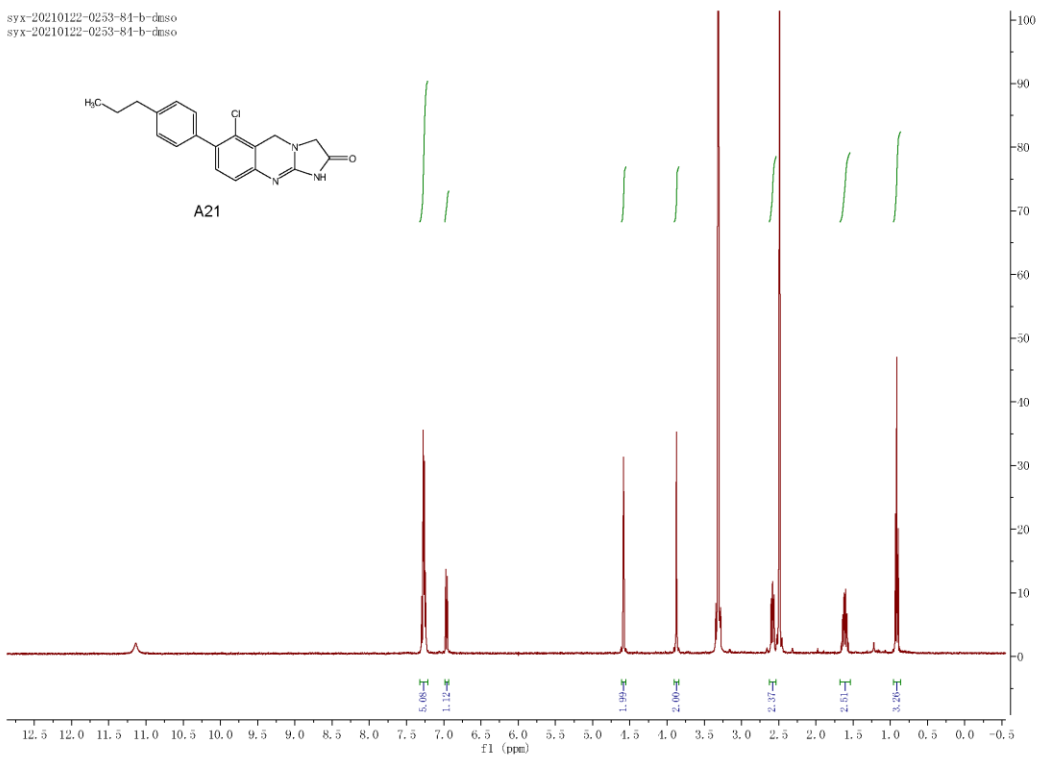
syx-20210122-0253-84-b-dmso
syx-20210122-0253-84-b-dmso
A21
5.08
1.12
1.99
2.00
2.37
2.51
3.26
f1 (ppm)

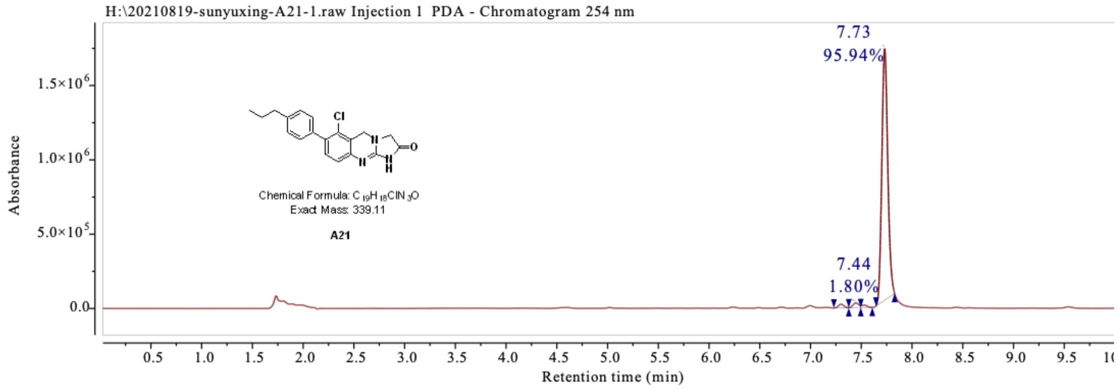
H:\20210819-sunyuxing-A21-1.raw Injection 1 PDA - Chromatogram 254 nm
7.73
95.94%
7.44
1.80%
Chemical Formula: $C_{19}H_{18}ClN_3O$
Exact Mass: 339.11
A21
Absorbance
Retention time (min)

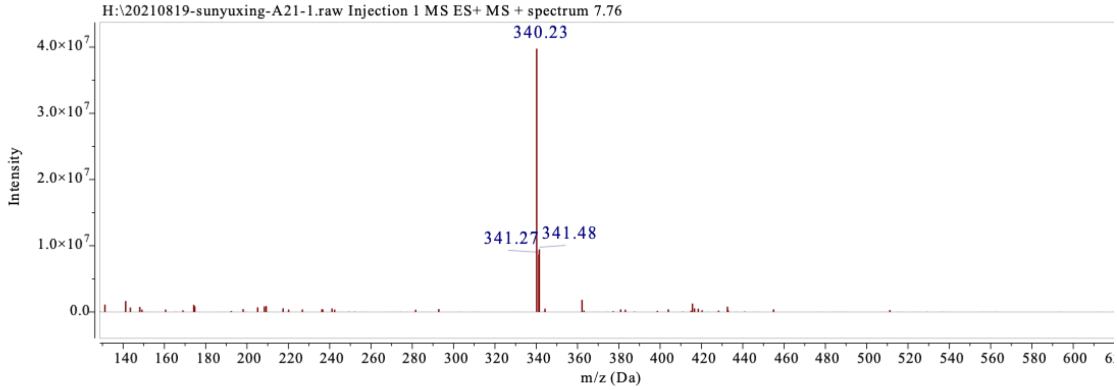
H:\20210819-sunyuxing-A21-1.raw Injection 1 MS ES+ MS + spectrum 7.76
340.23
341.27
341.48
Intensity
m/z (Da)

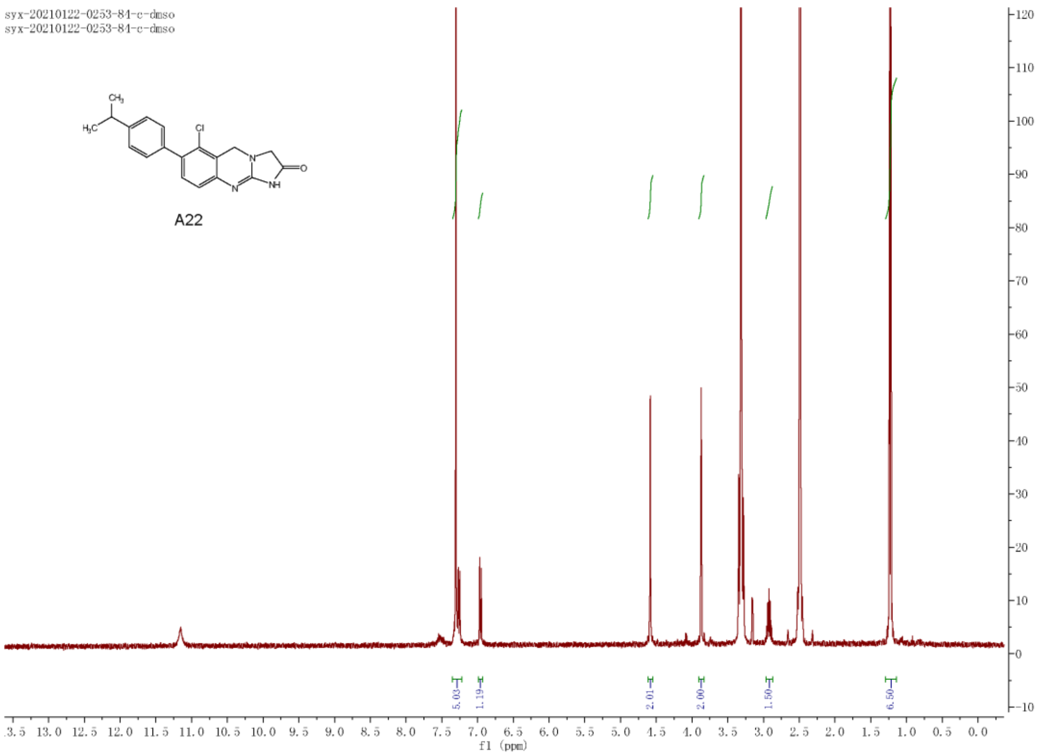
syx-20210122-0253-84-c-dmso
syx-20210122-0253-84-c-dmso
A22
5.03
1.19
2.01
2.00
1.50
6.50
f1 (ppm)

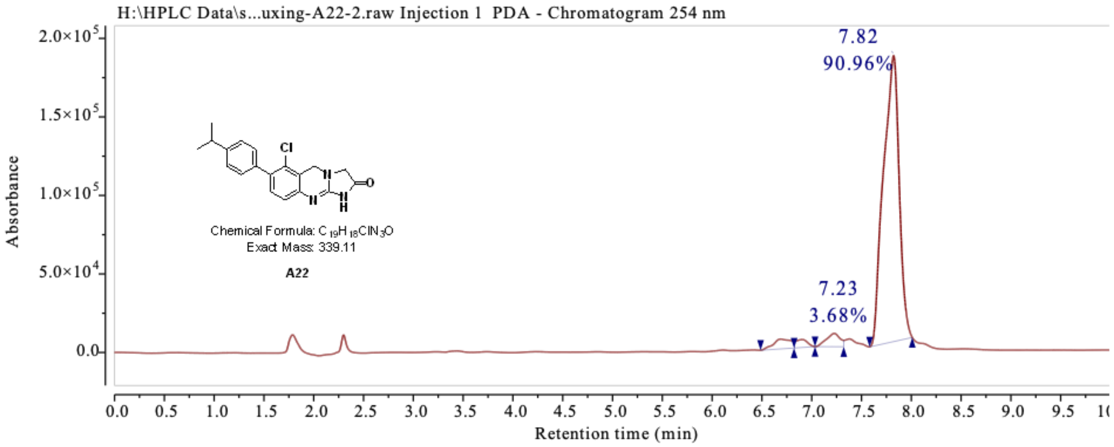
H:\HPLC Data\s...uxing-A22-2.raw Injection 1 PDA - Chromatogram 254 nm
Absorbance
Chemical Formula: $C_{19}H_{18}ClN_3O$
Exact Mass: 339.11
A22
7.82
90.96%
7.23
3.68%
Retention time (min)

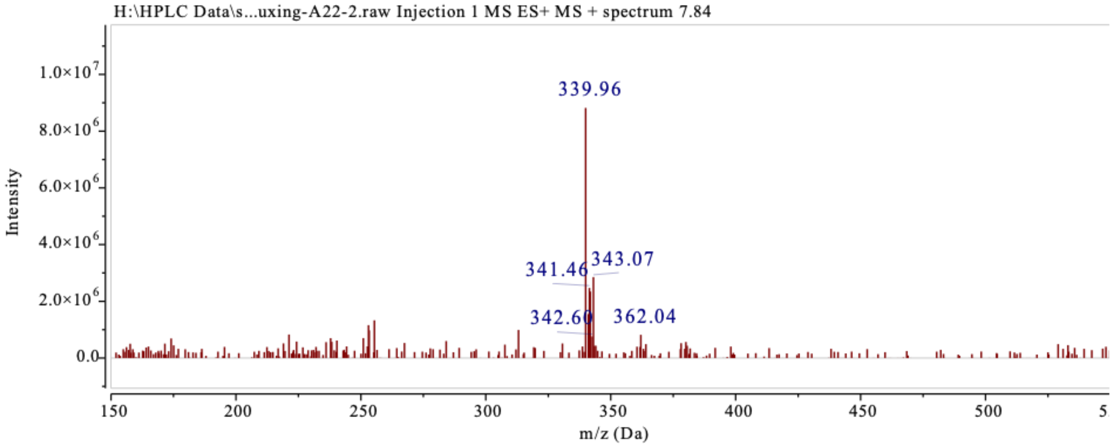
H:\HPLC Data\s...uxing-A22-2.raw Injection 1 MS ES+ MS + spectrum 7.84
Intensity
339.96
341.46
343.07
342.60
362.04
m/z (Da)
