# Supplementary material for: Structure of PDE3A–SLFN12 complex and structure-based design for a potent apoptosis inducer of tumor cells
Source: Nat Commun. 2021 Oct 27;12:6204. doi: 10.1038/s41467-021-26546-8 (PMC8551160; doi:10.1038/s41467-021-26546-8)
Supplement: Supplementary file 3 — Reporting summary [file 41467_2021_26546_MOESM3_ESM.pdf]

nature research

Corresponding author(s): Xiaodong Wang
Xiangbing Qi
Niu Huang
Hongwei Wang

Last updated by author(s): Sep. 22, 2021

Reporting Summary

Nature Research wishes to improve the reproducibility of the work that we publish. This form provides structure for consistency and transparency in reporting. For further information on Nature Research policies, see our Editorial Policies and the Editorial Policy Checklist.

Statistics

For all statistical analyses, confirm that the following items are present in the figure legend, table legend, main text, or Methods section.

n/a	Confirmed	
☐	☒	The exact sample size (n) for each experimental group/condition, given as a discrete number and unit of measurement
☐	☒	A statement on whether measurements were taken from distinct samples or whether the same sample was measured repeatedly
☐	☒	The statistical test(s) used AND whether they are one- or two-sided<br>*Only common tests should be described solely by name; describe more complex techniques in the Methods section.*
☒	☐	A description of all covariates tested
☒	☐	A description of any assumptions or corrections, such as tests of normality and adjustment for multiple comparisons
☐	☒	A full description of the statistical parameters including central tendency (e.g. means) or other basic estimates (e.g. regression coefficient) AND variation (e.g. standard deviation) or associated estimates of uncertainty (e.g. confidence intervals)
☐	☒	For null hypothesis testing, the test statistic (e.g. F, t, r) with confidence intervals, effect sizes, degrees of freedom and P value noted<br>*Give P values as exact values whenever suitable.*
☒	☐	For Bayesian analysis, information on the choice of priors and Markov chain Monte Carlo settings
☒	☐	For hierarchical and complex designs, identification of the appropriate level for tests and full reporting of outcomes
☒	☐	Estimates of effect sizes (e.g. Cohen's d, Pearson's r), indicating how they were calculated

Our web collection on statistics for biologists contains articles on many of the points above.

Software and code

Policy information about availability of computer code

Data collection	We used Tecan GENios microplate reader for cell viability determination. We used OpenGrowth (version 1.0.1) and Protein Local Optimization Program (PLOP) (version 25.1) for molecular glue design and evaluation. We used AutoEMation software to collect cryo-EM datasets, written by Dr. Jianlin Lei at Tsinghua University.
Data analysis	We used GraphPad Prism 8.0.1 and Microsoft office home and student 2019 for statistical analysis. We used MotionCor2 (version 1.1.0) to correct the beam-induced motion of cryo-EM micrographs and used Relion (version 3.1.3) to perform 3D reconstruction. The CTF values of these motion-corrected micrographs were determined by CTFFIND4 algorithm (version 4.15). The atomic models were firstly generated by SWISS-MODEL (web server: https://swissmodel.expasy.org) and manually built in COOT (version 0.9.6), and further real-space refined in Phenix (version 1.19.2). The structural analysis was performed in UCSF Chimera (version 1.13.1). All these softwares are open-source.

For manuscripts utilizing custom algorithms or software that are central to the research but not yet described in published literature, software must be made available to editors and reviewers. We strongly encourage code deposition in a community repository (e.g. GitHub). See the Nature Research guidelines for submitting code & software for further information.

Data

Policy information about availability of data

All manuscripts must include a data availability statement. This statement should provide the following information, where applicable:

- Accession codes, unique identifiers, or web links for publicly available datasets
- A list of figures that have associated raw data
- A description of any restrictions on data availability

Data supporting our findings in this manuscript are available from the corresponding authors upon reasonable request. Raw blots corresponding to the SDS-PAGE gels, cell viability, chemistry synthesis and tumor xenograft are included as Source Data file. The cryo-EM maps of anagrelide-, DNMDP-, and nauclefine-induced

PDE3A-SLFN12 complexes have been deposited in the EMDB under accession number EMD-31103, EMD-31104, and EMD-31105, respectively, and the coordinates in the PDB with PDB ID: 7EG0 (https://doi.org/10.2210/pdb7EG0/pdb), 7EG1 (https://doi.org/10.2210/pdb7EG1/pdb) and 7EG4 (https://doi.org/10.2210/pdb7EG4/pdb), respectively. Source data are provided with this paper.

Field-specific reporting

Please select the one below that is the best fit for your research. If you are not sure, read the appropriate sections before making your selection.

☒ Life sciences ☐ Behavioural & social sciences ☐ Ecological, evolutionary & environmental sciences

For a reference copy of the document with all sections, see nature.com/documents/nr-reporting-summary-flat.pdf

Life sciences study design

All studies must disclose on these points even when the disclosure is negative.

Sample size	The cell viability assays were determined with 3 wells each sample to perform statistical testing. Gels were repeated for 2 times. Sarmple size of each group of mice was n=5. For cryo-EM analysis particle numbers used for the final reconstructions were 82,930 for anagrelide-induced complex, 203,914 for DNMDP-induced complex, and 60,683 for the nauclefine-induced complex. The resolutions of the structures of anagrelide-, DNMDP- and nauclefine-induced PDE3A-SLFN12 complex were 3.4-Å, 3.2-Å and 3.2-Å resolution, respectively, estimated by the Fourier Shell Correction (FSC)=0.143 cutoff criteria.
Data exclusions	For cryo-EM reconstruction, particles grouped in bad classes with poorly defined features were excluded, because these particles were normally denatured or dissociated samples, which were harmful for high-resolution 3D reconstruction.
Replication	Experiments that led to quantitative conclusions were repeated (as indicated in figure legends).
Randomization	Samples were allocated random. For the animal experiments, we used the same age and sex of mice.
Blinding	For the mouse xenograft experiments, human HeLa cells were subcutaneously injected into nude mice, then the mice were randomly divided into 4 groups for the treatment with vehicle and compounds. For cryo-EM reconstruction, particles were randomly divided into two parts, and used for 3D structure determination. The consistence of structures generated by these two sub-datasets was used for the blinding test.

Reporting for specific materials, systems and methods

We require information from authors about some types of materials, experimental systems and methods used in many studies. Here, indicate whether each material, system or method listed is relevant to your study. If you are not sure if a list item applies to your research, read the appropriate section before selecting a response.

Materials & experimental systems

n/a	Involved in the study	
☐	☒	Antibodies
☐	☒	Eukaryotic cell lines
☒	☐	Palaeontology and archaeology
☐	☒	Animals and other organisms
☒	☐	Human research participants
☒	☐	Clinical data
☒	☐	Dual use research of concern

Methods

n/a	Involved in the study	
☒	☐	ChIP-seq
☒	☐	Flow cytometry
☒	☐	MRI-based neuroimaging

Antibodies

Antibodies used	PDE3A antibody from Bethyl Laboratories (1:1000, Cat# A302-740A); anti-Rabbit-HRP antibody from Sigma-Aldrich (1:5000, Cat# A0545); anti-myc-HRP antibody from MBL (1:10,000, Cat#M047-7, clone PL14); anti Mouse-HRP antibody from Sigma-Aldrich (1:5000, Cat# A9044); Flag-HRP antibody from Sigma-Aldrich (1:10,000, Cat#A8592); anti-GAPDH-HRP antibody from MBL (1:50,000, Cat# M171-7,clone 3H12); anti-Actin-HRP antibody from MBL (1:50,000, Cat# PM053-7); SLFN12 antibody from abcam (1:1000, Cat#ab234418, PR20904-32E)
Validation	PDE3A antibody is suitable for WB and IP, reacts with human; SLFN12 antibody is suitable for WB and IHC-P, reacts with human; All the antibodies used in this study are commercially available and validated for their use as indicated according to the manufacturer's website. These antibodies are further validated and routinely used in our lab and all the detected-bands were matched with the predicted molecular weight.

Eukaryotic cell lines

Policy information about cell lines

Cell line source(s)	HeLa, HEK293T cell lines have been obtained from the American Type Culture Collection (ATCC).
Authentication	The cell lines were obtained from commercial source and none of the lines used were authenticated
Mycoplasma contamination	Cell lines used in this study were negative to mycoplasma by detection by PCR.
Commonly misidentified lines (See ICLAC register)	No commonly misidentified cell lines were used in this study

Animals and other organisms

Policy information about studies involving animals; ARRIVE guidelines recommended for reporting animal research

Laboratory animals	Female BALB/c athymic (nu/nu) nude mice (age, 4 weeks; weight, 18-20 g) were maintained in an animal facility with 12 h light/12 h dark cycles, temperature (22–24 °C), humidity (40–60%) at the National Institute of Biological Sciences, Beijing.
Wild animals	None
Field-collected samples	The study did not involve samples collected from the field.
Ethics oversight	Animal experimentation: Animal care and use followed the institutional guidelines of the National Institute of Biological Sciences (NIBS), Beijing (Approval ID: NIBSLuoM15C), and the Regulations for the Administration of Affairs Concerning Experimental Animals of China.

Note that full information on the approval of the study protocol must also be provided in the manuscript.
